# Toll-Like Receptors Drive Specific Patterns of Tolerance and Training on Restimulation of Macrophages

**DOI:** 10.3389/fimmu.2018.00933

**Published:** 2018-05-14

**Authors:** Suzanne K. Butcher, Christine E. O’Carroll, Christine A. Wells, Ruaidhrí J. Carmody

**Affiliations:** ^1^Centre for Stem Cell Systems, Faculty of Medicine, Dentistry and Health Sciences, University of Melbourne, Melbourne, VIC, Australia; ^2^Alimentary Pharmabiotic Centre, University College Cork, Cork, Ireland; ^3^Centre for Immunobiology, Institute of Infection, Immunity & Inflammation, College of Medicine, Veterinary and Life Sciences, University of Glasgow, Glasgow, United Kingdom

**Keywords:** tolerance, macrophage, toll-like receptor, transcriptome, innate immune memory, NF-κB

## Abstract

Tolerance is a long-recognized property of macrophages that leads to an altered response to repeated or chronic exposure to endotoxin. The physiological role of tolerance is to limit the potential damage to host tissue that may otherwise result from prolonged production of pro-inflammatory cytokines. Tolerance is induced by all toll-like receptor (TLR) ligands tested to date, however, tolerance induced by the TLR4 ligand lipopolysaccharide (LPS) is by far the best studied. LPS tolerance involves a global transcriptional shift from a pro-inflammatory response toward one characterized by the expression of anti-inflammatory and pro-resolution factors. Although largely reversible, LPS-tolerance leads to a hybrid macrophage activation state that is pro-inflammatory in nature, but possesses distinct regulatory anti-inflammatory features. Remarkably, a comparative transcriptomic analysis of tolerance induced by different TLR ligands has not previously been reported. Here, we describe the transcriptomic profiles of mouse macrophages tolerized with ligands for TLR2, TLR3, TLR4 and TLR 9. While we identified TLR-specific transcriptional profiles in macrophages tolerized with each ligand, tolerance induced by TLR4 represented an archetype pattern, such that each gene tolerized by any of the TLRs tested was also found to be tolerized by TLR4. Pro-inflammatory cytokines are not universally suppressed in all tolerant cells, but distinct patterns of cytokine expression distinguished TLR-specific tolerance. Analysis of gene regulatory regions revealed specific DNA sequence motifs associated with distinct states of TLR tolerance, implicating previously identified as well as novel transcriptional regulators of tolerance in macrophages. These data provide a basis for the future exploitation of TLR-specific tolerant states to achieve therapeutic re-programming of the innate immune response.

## Introduction

Innate immunity is the first line of host defense against infection and is critical for the development of adaptive immunity. Toll-like receptors (TLRs) are key sensors in the innate immune system and recognize conserved structures of microbial-derived molecules or pathogen associated molecular patterns (PAMPs). To date, 13 TLRs have been identified in mammals. These form homo- or heterodimers to recognize a range of PAMPs that spans microbial diversity, and regulation of this TLR repertoire fundamentally alters the tissue response to infection [reviewed in Ref. (1, 2)]. TLR activation induces the expression of hundreds of genes that encode inflammatory cytokines, type I interferons, antimicrobial proteins, and regulators of metabolism and regeneration; these molecules in turn mediate inflammation, anti-microbial immunity, and tissue regeneration.

The activation of different TLRs leads to specific transcriptional responses, *via* highly evolutionarily conserved signaling pathways. These are dependent on the adapter proteins, such as MyD88, TRIF, TIRAP, and TRAM, which direct activation of the NF-κB, MAPK, and IRF pathways (3). The combination of adapter proteins engaged by specific TLRs shapes the subsequent transcriptional and immune responses to the initiating ligand. The choice of adapter can be mediated by subcellular location of the TLR-pathogen engagement, with some intracellular (endosomal) TLRs preferentially signaling *via* non-MyD88 pathways, notably, TLR3 and TLR4. The synergistic activation of NF-κB, MAPK, and IRF pathways are important for activation of acute cytokine responses, including TNF, IL6, and IL1β.

The negative regulation of TLR-signaling events is critical to ensure that prolonged or repeated exposure to TLR ligands does not lead to uncontrolled or inappropriate inflammation and consequent damage to host tissue. The most important mechanism for controlling TLR activation is a form of tolerance to repeated exposure to a TLR ligand. This has been best described for lipopolysaccharide (LPS) activation of TLR4 and is otherwise known as endotoxin tolerance (4, 5). TLR-tolerance can be described as a state of altered responsiveness of cells to the repeated or chronic activation of TLRs, and includes the phenomena of cross tolerance, where pre-exposure to one TLR-ligand will reduce inflammatory responses to another (6, 7). TLR tolerance is observed in a number of cell types, predominantly monocytes, macrophages, and dendritic cells. A number of factors that promote LPS tolerance have been identified, including negative regulators of TLR4 signal transduction, such as IRAK-M (8), negative regulators of NF-ĸB-directed transcription, such as BCL-3 and NF-ĸB p50 (9), as well as the aryl hydrocarbon receptor and tryptophan catabolism (10). While convergent signaling *via* NF-κB is essential for acquisition of LPS-tolerance, it is not known how generalizable this may be to other TLR-ligands (6, 9). Chromatin changes at tolerized genes allow for persistence of altered responsiveness to re-stimulation of cells, but these changes are reversible over time, or in response to competing signals (11, 12).

Previous transcriptomic analysis of LPS (TLR4) tolerant cells identified two classes of TLR4-inducible genes; (i) tolerizable genes which are repressed during LPS tolerance, and (ii) non-tolerizable genes, which are not (11, 13, 14). The functional classification of LPS-inducible genes revealed that pro-inflammatory factors fall predominantly into the tolerizable class of genes, while genes which code for anti-microbial factors, including anti-microbial peptides and scavenger receptors, fall into the non-tolerizable class of genes. Thus, LPS tolerance represents a global transcriptional shift from a pro-inflammatory to a pro-resolution and anti-inflammatory response, while maintaining protective innate immune functionality in the context of chronic or continuing infection. Whether genes are tolerized or not likely reflects the impact of continued expression in the context of an inflammatory response and whether repression would be advantageous or deleterious. Furthermore, LPS tolerance is a transient state that allows cells to re-express pro-inflammatory factors in response to TLR ligands over time. Our previous transcriptomic analysis demonstrated that macrophages that have recovered from a tolerant state adopt a hybrid polarization state with features of both M1 and M2 macrophages (14).

To date, global transcriptomic analysis has only been performed for tolerance induced by LPS, and the similarity to tolerance induced by ligands for other TLRs is not known. In this study, we perform a comparative transcriptomic analysis of murine bone marrow-derived macrophages (BMDMs) tolerized with ligands for TLR2, TLR3, TLR4, and TLR9. Our analysis identifies a core set of genes tolerized by all TLR ligands tested, and further reveals a pattern of LPS-TLR4 tolerance that encompasses the patterns observed in TLR2, TLR3, and TLR9-tolerant states. We identified additional patterns of super-repression and super-induction on re-stimulation that indicate a diverse set of transcriptional events that shape the long-term response of macrophages to infection.

## Materials and Methods

### Murine Bone Marrow Isolation

Bone marrow was isolated from 6- to 8-week-old female C57BL/6 for generation of primary BMDM *in vitro*. Mice were sacrificed according to the Code of Practice for the Humane Killing of Animals under Schedule 1 to the UK Animals (Scientific Procedures) Act 1986, with procedures approved by the University College Cork Animal Experimentation Ethics Committee. Excess tissue was removed from the femur and tibia bones and then cleaned in sterile phosphate buffered saline (PBS) and 70% ethanol. Using a 21-gauge needle and syringe, bone marrow was isolated by flushing ice cold sterile PBS through the femur and tibia bones. Isolated bone marrow was re-suspended to generate a single cell suspension and passed through a 70 µM cell strainer to remove any debris. The bone marrow suspension was washed twice in culture media (DMEM, 10% FBS, 1% penicillin/streptomycin, 1% l-glutamine, 1% non-essential amino acids), and centrifuged at 4°C at 300 × *g* for 5 min. Bone marrow was cryopreserved in fetal calf serum supplemented with 10% DMSO until required for use. Each biological replicate was derived from a different mouse.

### BMDM Differentiation

Bone marrow was cultured following isolation or from cryopreserved stocks in culture media supplemented with 30% L929 conditioned media for 7 days. The cells were cultured on sterile non-tissue culture-treated petri dishes. On day 3, BMDM differentiation media was removed and replaced with fresh media supplemented with 30% L929 conditioned media and any non-adherent cells removed. Differentiated BMDMs were removed from the petri dishes at day 7 by incubating the cells with 5 mM EDTA in sterile PBS at 37°C for 5 min. Cells were washed twice in culture media at 4°C for 5 min at 300 *g*, re-suspended in media without L929 conditioned medium, transferred to tissue culture-treated dishes and allowed to adhere overnight. The purity of BMDMs was assessed by flow cytometry and was typically greater than 95% F4/80 positive.

### TLR Tolerance

**Figure 1:**
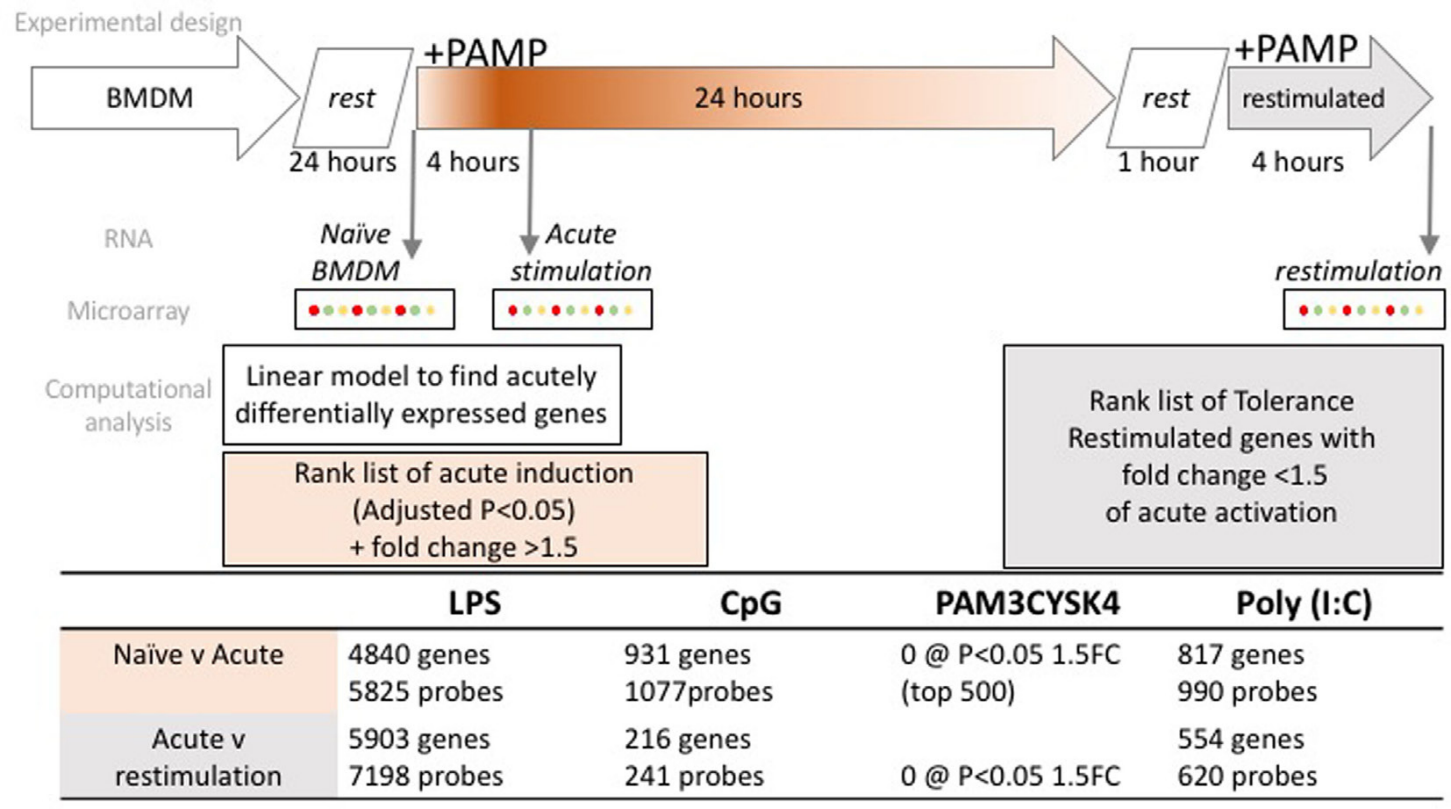
Experimental overview and workflow for transcriptome data normalization, filtering, and analysis. Bone marrow derived macrophages (BMDM) were differentiated for 7 days in L-cell conditioned media, washed and rested for 24 h, prior to acute stimulation with one of four toll-like receptor ligands. Cells were washed after 24 h, rested for 1 h, prior to restimulation with the same ligand. RNA was collected as indicated, from naïve, 4 h (acute) and 4 h (restimulated) time points. Microarray profiling followed, with the numbers of microarray probes, and correspondingly annotated genes that met the criteria for acute induction, or tolerance, as indicated in the table.

Toll-like receptor tolerance was induced in BMDMs by stimulating cells for 24 h with 100 ng/ml LPS (Invivogen), 100 ng/ml Pam3CSK4 (Invivogen), 10 µg/ml Poly(I:C) (GE Healthcare), or 1 µM CpG (1,826 sequence, Eurofins). These concentrations of TLR ligands have previously been established to be appropriate for the induction of a robust inflammatory response (9, 14). After 24 h, the media was removed and the cells were washed twice with sterile PBS. The cells were allowed to rest in fresh culture media for 1 h before a second stimulation for 4 h with the same TLR ligand (Figure 1).

### Immunoblotting

Whole-cell proteins were extracted using RIPA lysis buffer supplemented with protease inhibitors (50 mM Tris–HCl, pH 7.4, 1% Nonidet P-40, 0.25% SDS, 150 mM NaCl, 1 mM EDTA, 1mM PMSF, 1mM NaF, 1mM Na_3_VO_4_, 2 µg/ml aprotinin, 1 µg/ml pepstatin, and 1 µg/ml leupeptin). Lysates were resolved on SDS-PAGE gels, transferred to nitrocellulose membranes and immunoblotted with anti-p105/p50 (Cell Signaling Technology), anti-BCL-3 (AbCam).

### Gene Expression Analysis

Total RNA was isolated using the RNeasy mini kit (Qiagen) with all samples DNase treated according to manufacturer’s instructions. Triplicate biological replicate samples submitted for microarray profiling met all sample submission criteria (Beckman Coulter Genomics, NC, USA). Briefly, 200 ng of total RNA was fluorescently labeled with Cy3 nucleotides. Labeled RNA (cRNA) was hybridized to Agilent mouse 8 × 60 K microarrays (Agilent-028005). Each BMDM culture was generated from bone marrow pooled from three mice, and each pool generated independently for each replicate. Real-time PCR (qPCR) was performed using the universal probe library system (Roche) and primer sequences as follows; *Il6* forward 5′-tctaattcatatcttcaaccaagagg-3′ *Il6* reverse 5′-tggtccttagccactccttc-3′; *Tnf* forward 5′-tcttctcattcctgcttgtgg-3′ *Tnf* reverse 5′-ggtctgggccatagaactga-3′ 18s forward 5′-aaatcagttatggttcctttggtc-3′; 18s reverse 5′-gctctagaattaccacagttatccaa-3′. Relative mRNA levels were calculated using the ΔΔCT method.

Microarray data were processed as follows: data were normalized using Limma (3.26.9), including RMA background correction quantile normalization (15). Analysis workflow is described in Figure 1: briefly, only probes mapping to an ENSEMBL (v67) gene were retained for this analysis. A detection threshold was applied to remove probes that were not expressed in at least 2/3 replicates. A linear model was fitted to identify differentially expressed probes using an adjusted *p*-value (Benjamini and Hochberg) of 0.05. A tolerized gene was defined by the following two criteria: where the same probe was (a) significantly induced (*p* < 0.05) and exhibited 1.5-fold or more inducible expression in response to the first stimulation, then (b) 1.5-fold lower induction in the re-stimulated condition. Tolerized probes were grouped by partitioning around medoids (PAM) clustering [R “cluster” package (v2.0.6)] (16). Functional enrichment analysis was conducted on the top 500 differentially expressed genes in each condition, ranked but not filtered on *p*-value. Enriched pathways and GO terms were identified using the curated data at InnateDB (17), and protein–protein interactions were identified using STRINGDB (18). Transcription factor motif enrichment was identified using Hypergeometric Optimization of Motif EnRichment [HOMER (v4.8.3)] (19). Heatmaps were generated using a similarity metric derived from the Pearson correlation, or using the www.stemformatics.org hierarchical clustering tool. Raw data are available from GEO (GSE81291) and www.stemformatics.org (20) and processed data can be visualized at http://www.stemformatics.org/datasets/search?ds_id=6943.

## Results

### A Comparative Analysis of TLR Tolerance Identifies TLR4 Induced Tolerance as the Dominant Form

Lipopolysaccharide is classically used to study macrophage tolerance. Although tolerance may be induced by TLRs other than TLR4, a comparative transcriptomic analysis of tolerance induced by different TLRs has not previously been performed. The similarity between transcriptional responses in macrophages tolerized by ligands for TLR2, TLR3, TLR4, and TLR9 following re-stimulation was assessed in mouse BMDM stimulated with Pam3CSK4 (TLR2), Poly(I:C) (TLR3), LPS (TLR4), and CpG DNA (TLR9). 24 h following stimulation the cells were washed then re-stimulated with the same TLR ligand for an additional 4 h, prior to RNA isolation and microarray analysis. A subset of these patterns was confirmed by qPCR.

**Figure 2:**
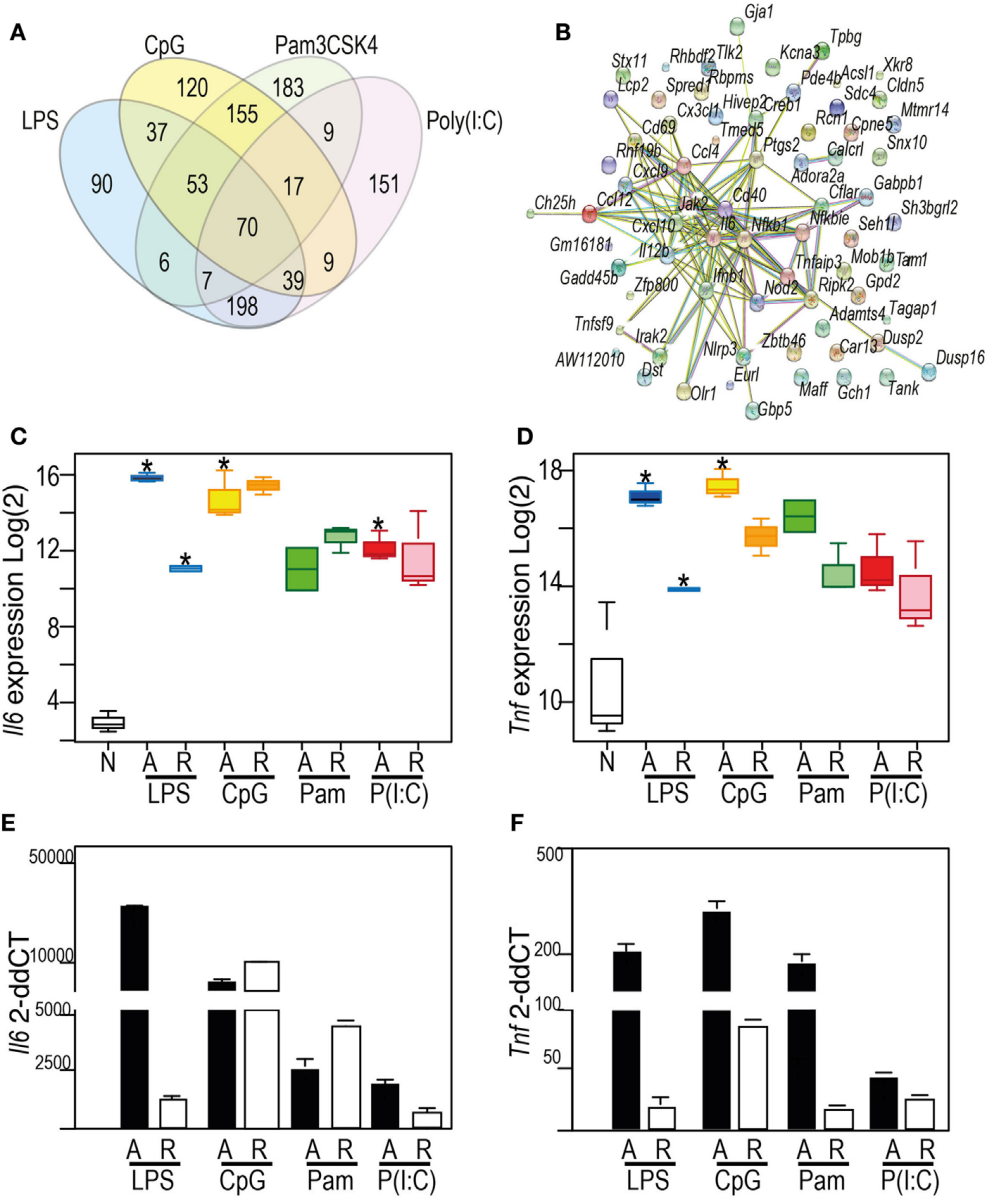
Toll-like receptor (TLR) ligands acutely activate macrophages, with shared classes of inflammatory response **(A)** Venn diagram showing the overlap of the top 500 inducible genes in the acute (4 h) response to: 100 ng/ml lipopolysaccharide (blue), 1 µM CpG (yellow), 100 ng/ml Pam3CSK4 (green), 10 µg/ml Poly(I:C) (red). **(B)** Protein–protein interaction network derived from StringDB for the proteins encoded by the 70 genes commonly induced by the four TLR ligands indicated in Venn. Nodes: proteins. Edges: interactions from STRING database. **(C,D)** Patterns of gene expression from the microarray data for *Il6* **(C)** and *Tnf* **(D)** mRNA. *Y*-axis (Log_2_) expression; *X*-axis showing naïve (N), acute (A), or re-stimulated (R) bone marrow-derived macrophages (BMDM). **(E,F)** qRT-PCR measurement of *Il6* **(E)** or *Tnf* **(F)** mRNA. *Y*-axis: microarray (Log_2_) expression. For Box-whisker plots, median, min, max shown, *n* = 2 or 3 samples * Benjamini and Hochberg adjusted *p* < 0.05. For histograms, delta–delta CT normalized to normalized to naïve BMDM, *n* = 3. See also Supplementary Figure 1: TF motif analysis of top 500 DE genes.

We first confirmed that all four stimuli activated BMDM that had received no prior stimulus, and that this activation profile was consistent with known TLR responses in macrophages (Figure 2; Figure S1 and Table S1 in Supplementary Material). We compared the top 500 genes induced by each ligand (Figure 2A), and observed a robust activation profile for known inflammatory markers, including IL6 (Figure 2C) and TNF (Figure 2D), confirming these patterns using qPCR (Figures 2E,F). The genes that were acutely induced in all conditions were predominantly chemokines and cytokines, known to be regulated by transcription factors NF-κB, MAPK, and IRF. These factors were themselves targets of TLR signaling and represented by hubs in a STRING protein network (Figure 2B). Although distinct patterns of gene expression are evident for each TLR ligand, functional enrichment of pathways and molecular processes (Table S2 in Supplementary Material) shows that these converge on similar biological processes.

Whereas NF-κB activation is a common theme to all the TLR ligands profiled here, the MAPK signaling pathways were more significantly enriched in the gene sets induced by CpG and Pam3CSK4, and type I interferon pathways were enriched in Poly(I:C) and LPS-driven gene sets. A set of 198 genes (Table S1 in Supplementary Material) upregulated by LPS and Poly(I:C), but not CpG and Pam3CSK4, was rich in genes which regulate, and are driven by type I interferons. This included positive regulators *Azi2* and *Isg15*; and genes for IRF3/7-activating DNA sensors cGAS (*E330016A19Rik*), ZBP1, RIG-I (*Dhx58*), and MDA5 (*Ifih1*). Negative regulators of type I interferon signaling were also induced, including *Nlrc5* and *Usp18*. LPS and Poly(I:C)-driven gene sets were significantly enriched for transcription factor motifs for IRF family members (Figure S1 in Supplementary Material).

**Figure 3:**
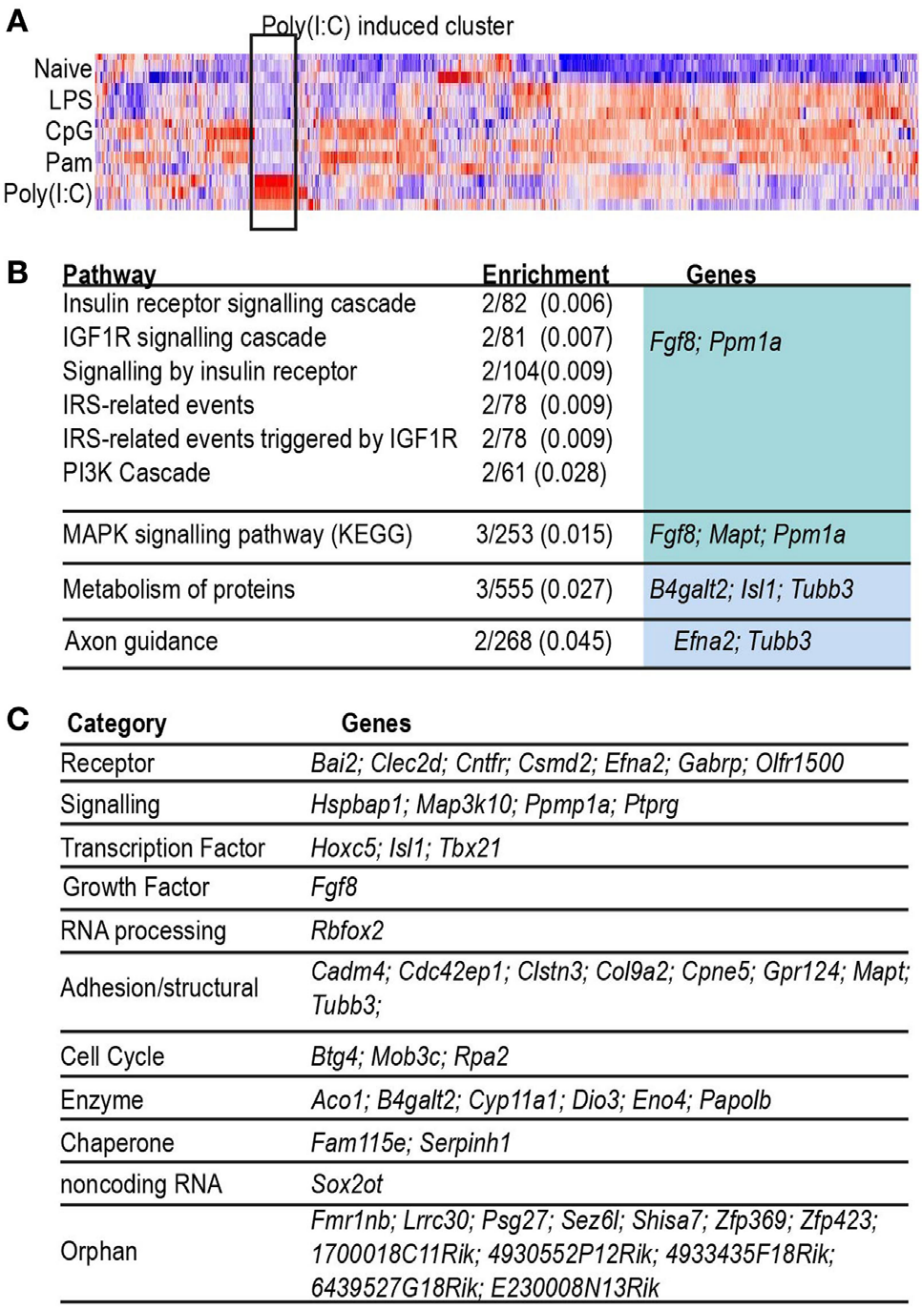
Characterization of probes induced only by Poly(I:C). **(A)** Heat map (Pearson correlation) of combined list of top 500 acutely induced genes per acute condition. Color indicates row *z*-score, ranging from −2 (blue) to 2 (red). Highlight box indicates cluster of probes highly induced only in Poly(I:C). **(B)** Table of Reactome pathways enriched (adjusted *p* < 0.05) in genes induced uniquely by acute infection with Poly(I:C). Enrichment: number of genes in test list/number of genes in pathway (adjusted *p*-value). **(C)** Table of genes grouped by biological category.

As can be seen in the Venn diagram in Figure 2A, 47% of the inducible gene set was ligand-specific, so we searched for evidence of gene groups whose engagement was restricted to activation of a single TLR. The heatmap shown in Figure 3 illustrates a group of 55 genes with highly correlated expression patterns, that were induced only in the acute Poly(I:C) condition. The pathway enrichment terms associated with this group of genes were largely driven by expression of *Fgf8* and *Ppm1a*, which are part of insulin, MAPK, and phosphoinositide 3-kinase signaling pathways. Additionally, genes in this group included candidates for anti-viral (*Papolb*) and immune signaling activities (*Ppm1a, Trim12c, Csmd2*, and *Tbx21*). However, few of the 55 genes have been characterized extensively in an immune context, and 15% of this gene set consisted of genes uncharacterized in any context, highlighting the potential for further characterization of the TLR3-induced transcriptome.

**Figure 4:**
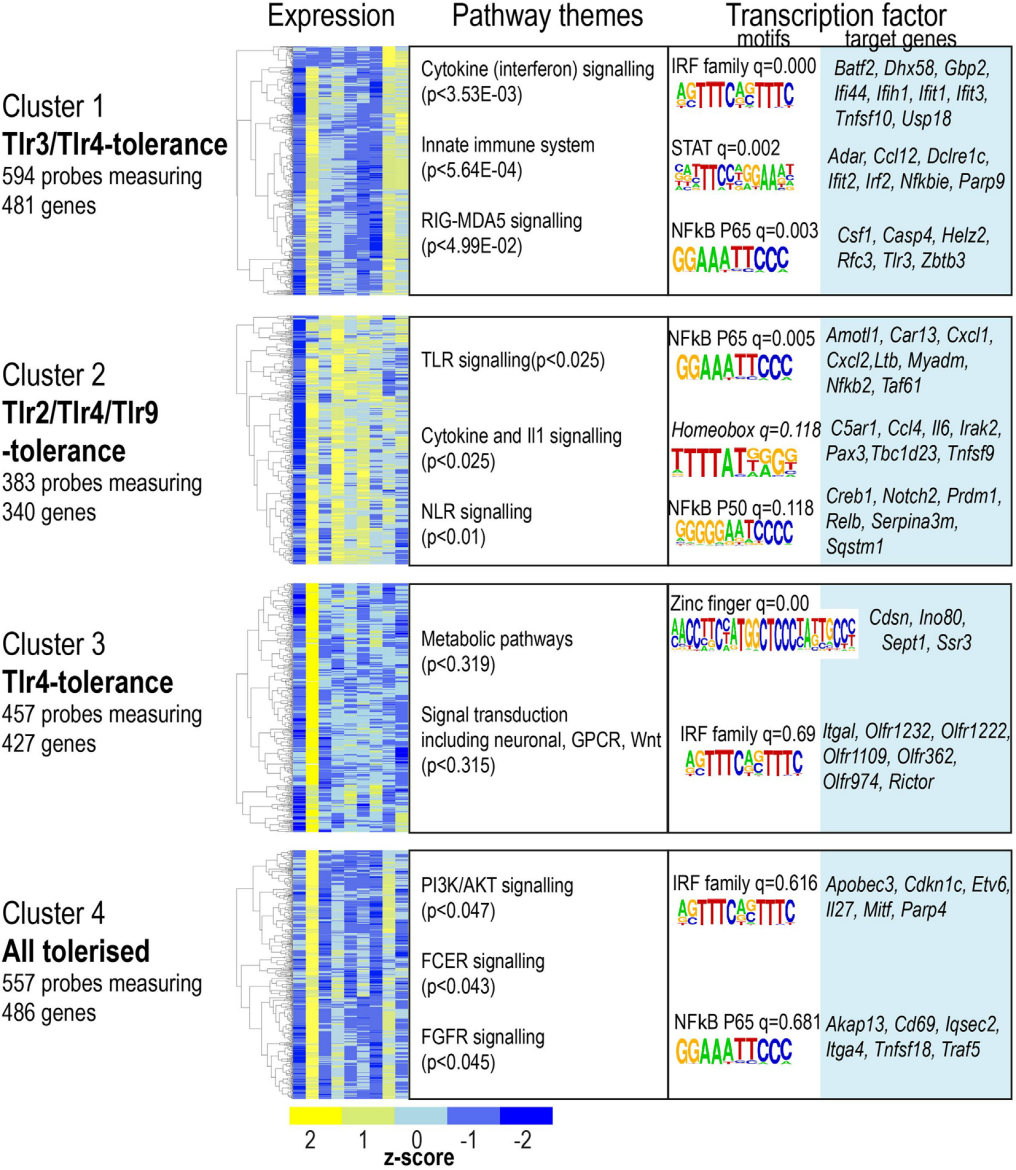
Four clusters of gene expression reveal toll-like receptor-specific patterns of tolerance. Microarray probes showing tolerance in at least one infection were grouped using partitioning around medoids-clustering. Heat maps for each cluster. Color indicates row *z*-score of mean log_2_ expression. Columns (L–R) naïve, lipopolysaccharide acute, and restimulation; CpG acute, restimulation; Pam3csk4 acute, restimulation; Poly(I:C) acute and restimulation. Pearson correlation of *z* scores shown in rows. Significantly enriched pathways, grouped thematically. Maximum adjusted *p*-value for all pathways significantly enriched in that theme is shown. Transcription factor binding motifs enriched in each cluster. Motif logos and adjusted *q*-values are representative for each transcription factor family. Full motif enrichment results are available at www.stemformatics.org.

Tolerized genes were defined as those significantly upregulated at 4 h acute stimulation, but demonstrating at least 1.5-fold lower activation on re-stimulation. A total of 1,644 genes matched this pattern in one or more condition (Table S1 in Supplementary Material). We used PAM clustering to identify four groups of genes with shared patterns of induction, or tolerance (Figure 4, referred to as gene “clusters”). The largest group of tolerized genes was a pattern common to LPS and Poly(I:C) (Figure 4, cluster 1); in contrast, genes that were tolerized in all conditions except Poly (I:C) represented the smallest group (Figure 4, cluster 2). A substantial subset of genes was only tolerized under LPS stimulation (Figure 4, cluster 3); and the remaining genes were tolerized in all conditions (Figure 4, cluster 4). Surprisingly, LPS-tolerance represents a group of tolerized-genes common to all other TLRs tested here, and genes tolerized by any TLR were subsets of this TLR4 tolerized pattern.

Given the high degree of overlap between the patterns of tolerance observed for LPS or Poly(I:C), we examined the promoter regions of genes in cluster 1 (Figure 4), and found that these were enriched for both NF-κB and IRF motifs. In contrast, the subset of genes in cluster 2 that were exclusively not tolerized by Poly(I:C) also lacked the IRF motif. These were not tolerized because they were not acutely upregulated in the Poly(I:C) condition. Likewise, the genes that were only tolerized by LPS (cluster 3) were not acutely induced in the other conditions. The promoter regions of this gene set were largely dominated by the presence of an IRF4 and zinc finger motif. NF-κB p65-Rel promoter motifs were common to the majority of tolerized clusters, an observation consistent with previous links between NF-κB and tolerance (21).

**Figure 5:**
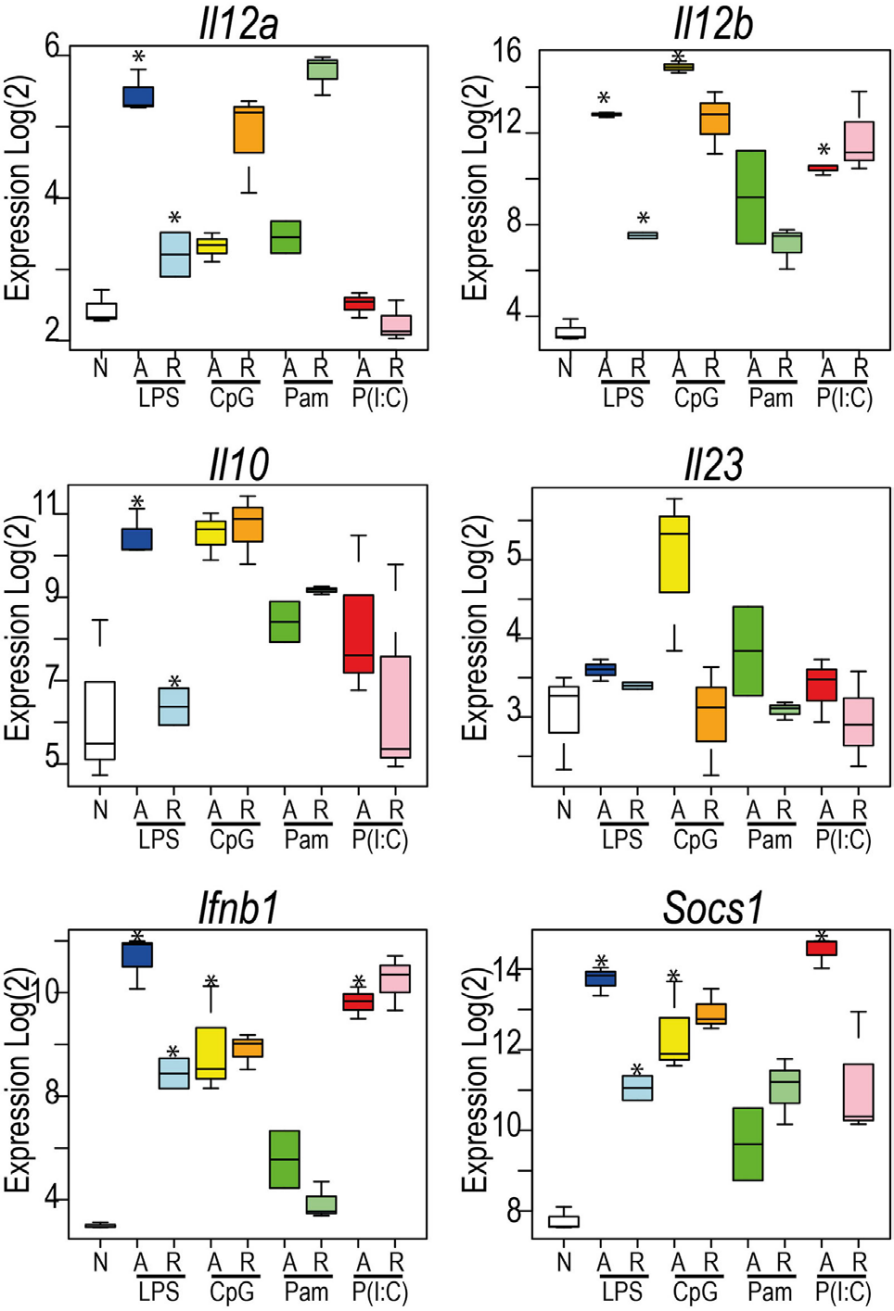
Toll-like receptors exhibit distinct patterns of tolerance. Microarray profiles of *Il12a, Il12b, Il23a, Ifnb1, Socs1*, and *Il10*. *Y*-axis: Log_2_ expression. *X*-axis: conditions. See also Figures S2–S4 in Supplementary Material. Box and whisker plots are median, min, and max. *n* = 2–3. *indicates Benjamini and Hochberg adjusted *p* < 0.05.

These data revealed the surprising observation that the genes identified in any TLR-tolerance state are also always found in LPS-tolerance, indicating that LPS/TLR4-tolerance represents the archetype tolerizable state in BMDM. While LPS represented the most comprehensively tolerized condition, significant differences were observed in the capacity of other TLRs to reduce pro-inflammatory cytokine and chemokine expression in the tolerant state. For example, while *Tnf* expression was consistently tolerized in all conditions, *Il6* was tolerized only in cells re-stimulated *via* LPS-TLR4 and Poly(I:C)-TLR3 (see Figure 2). Similarly, *Ifnb1* was tolerized by ligands for TLR4 and TLR2, but not by TLR3 or TLR9, while *Il10* was tolerized by ligand for TLR3 and TLR4, but not for TLR2 and TLR9 (Figure 5). IL-12 subunits were also differentially tolerized: *Il12a* was tolerized only in LPS conditions, whereas *Il12b* was tolerized by all conditions except Poly(I:C). These patterns are exemplified in Figure 5, and illustrated for TLR, NLR pathways, cytokines, growth factors, and chemokines in Figures S2–S4 in Supplementary Material.

#### Innate Memory—Super-Induction, Super-Repression, and Delayed Responsiveness to TLR Ligands

**Figure 6:**
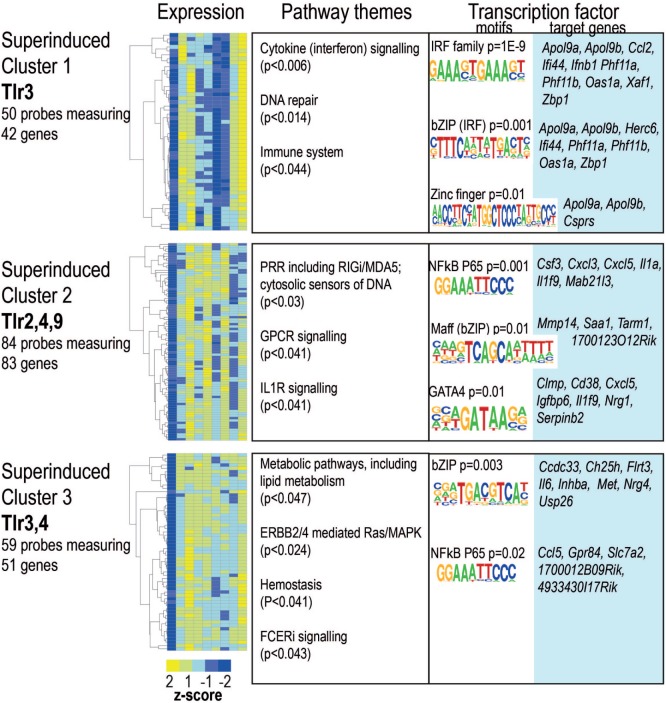
Ligand-specific patterns of super-induction on toll-like receptor (TLR) re-stimulation. Probes showing super-induction in at least one infection were clustered using partitioning around medoids. Heat maps for each cluster. Color indicates row *z*-score of mean log_2_ expression. Columns (L–R) naïve, lipopolysaccharide acute, and restimulation; CpG acute, restimulation; Pam3csk4 acute, restimulation; Poly(I:C) acute, restimulation. Pearson correlation of *z* scores shown in rows. Significantly enriched pathways, grouped thematically. Maximum adjusted *p*-value for all pathways significantly enriched in that theme is shown. Transcription factor binding motifs enriched in each cluster. Motif logos and *p*-values are representative for each transcription factor family. Full motif enrichment results are available at www.stemformatics.org.

By definition acute induction was a pre-requisite of tolerance, however, not all acutely induced genes were tolerized. Arguably, tolerance represents one form of innate “priming” or “training,” where pre-exposure to a PAMP alters macrophage responses to re-exposure. We identified 174 acutely induced genes that demonstrated further induction on re-stimulation with one or more TLR ligand (Figure 6; Table S1 in Supplementary Material). Over a third of these genes were predicted to be secreted factors, including chemokines *Ccl2, Ccl5, Ccl8, Cxcl3*, and *Cxcl5*; cytokines *Csf3, Ifnb1, Il1a, Il1b, Il6, Il12b*, and *Il18bp*. As evident from Figures S2–S4 in Supplementary Material, many of these were tolerized or super-induced depending on the pathway of activation. Again, the dominant patterns were shared between TLR4/LPS and TLR3/Poly(I:C), with enrichment of IRF, bZip, and NF-κB motifs in the promoters of these “super-induced” genes.

Genes that were transiently repressed, re-gaining at least 1.5-fold expression upon re-stimulation (Figure S5 and Table S1 in Supplementary Material) were dominated by genes involved in metabolic respiration, with motif enrichment implicating ETS factors as the major transcriptional regulator of this pattern. A smaller set of genes were acutely downregulated, then further strongly repressed on re-stimulation (Figure S6 and Table S1 in Supplementary Material). These were predominantly genes implicated in cell cycle processes, and may reflect the culture system (mouse BMDM).

**Figure 7:**
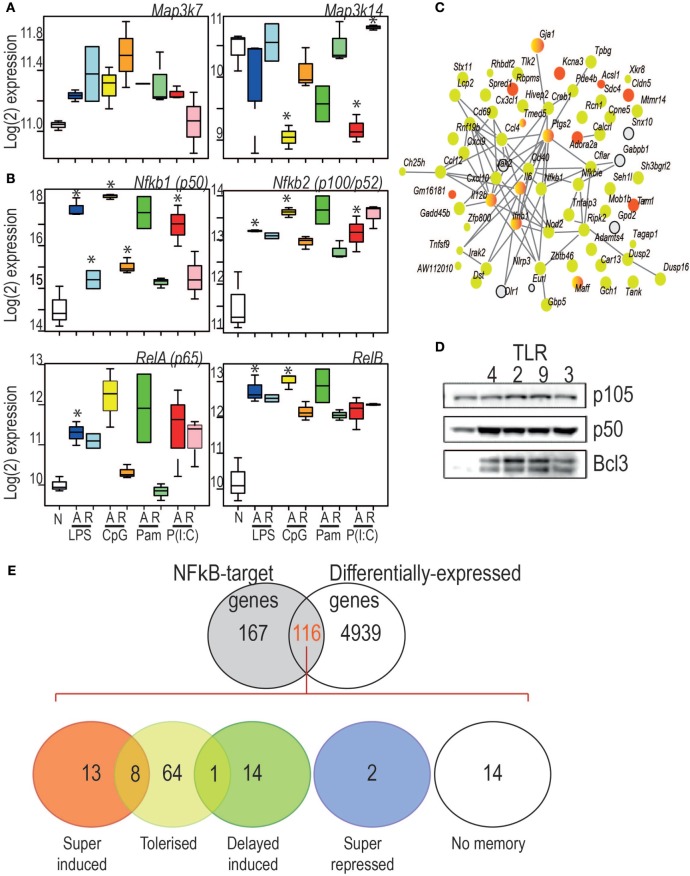
**(A)** Log_2_ expression of two kinases central to the canonical (left) and non-canonical (right) NF-κB pathways. **(B)** Key components of the canonical and non-canonical NF-κB pathways exhibit different regulatory patterns. Y-axis: Log_2_ expression. *indicates significant differential expression (adjusted *p* < 0.05). **(C)** StringDB protein–protein association network for the proteins encoded by the 70 genes commonly induced during acute responses by the four toll-like receptor ligands. Nodes: proteins. Edges protein–protein association. Colors: yellow: tolerized; red: super-induced; gray: no memory pattern. **(D)** Western Blot analysis of p105, p50, and BCL3 expression in cells stimulated for 24 h with ligand for TLR4, TLR2, TLR9, and TLR3 as indicated. **(E)** Characterization of differential expression and memory status of NF-κB target genes. Figure 7E overlap between NF-κB target genes and differentially expressed genes (adjusted *p* < 0.05, any acute infection). Number of genes per pattern is indicated in each colored circle. Circle overlaps show numbers of genes with both patterns, either due to multiple differently behaving probes, or ligand-specific memory pattern differences.

The set of 70 genes acutely induced in all conditions were overwhelmingly subjected to a tolerance pattern, with a small number exhibiting ligand-specific super-induction (Figure 7C). This may indicate that coordinate mechanisms determine whether the expression of an inflammatory mediator is tolerized or trained. Indeed, the core members of NF-κB activation were themselves subjected to altered expression on re-stimulation with a TLR-ligand (Figure 7A–B). Increased levels of the NF-κB p50 subunit and the IĸB protein BCL-3 were induced by all TLR ligands tested (Figure 7D) underlying the previously established role for the p50:BCL-3 transcriptional repressor complex in limiting TLR responses (9). NF-κB target genes were highly represented in BMDM genes with a “memory” status of the original stimulation, with the vast majority of these categorized in the tolerized group (Figure 7E).

## Discussion

Historically, the study of endotoxin tolerance was performed using LPSs. Early research on endotoxin tolerance *in vivo* relied upon the fever response as a readout for responsiveness to endotoxin and led to the concept that tolerance was a hyporesponsive state due to a desensitization to endotoxin. However, as the factors that mediate the innate inflammatory response were identified it became apparent that tolerance is a state of altered responsiveness to stimulation rather than simply hyporesponsiveness. Initial transcriptomic analysis of LPS-induced tolerance in macrophages underscored this and revealed that a large number of LPS-inducible genes, particularly those encoding anti-microbial, anti-inflammatory, and pro-resolution factors are not suppressed during LPS tolerance (11). Remarkably, the same transcriptomic analysis has not previously been applied to tolerance induced by ligands for other TLRs, and thus the relationship between tolerant states induced by specific TLRs remained unclear. In this study, we have addressed this by performing transcriptomic analysis of macrophages tolerized with ligands for TLR4, TLR2, TLR3, and TLR9. A comparative analysis of the transcriptomic profiles of TLR-specific tolerant cells provides us with fundamental insights into the molecular programming of the innate immune inflammatory response. Our data reveal that tolerance induced by each TLR is distinct and that TLR4 induced tolerance is the most comprehensive tolerant state relative to tolerance induced by other TLRs.

The original observations of endotoxin hyporesponsiveness were conducted using *in vivo* models. Here, we have removed the paracrine milieu of cytokines and growth factors that would contribute to sustained macrophage activation, as well as endothelial or T-cell derived factors that may polarize recruited leukocytes. Pretreatment of LPS-tolerized macrophages with GM-CSF or IFNγ can partially restore TNF production *in vivo* after a second LPS injection, although not to the levels seen in naïve mice (22). In the current study, we differentiated BMDM using L-cell conditioned media, but the stimulation of cells was undertaken in the absence of growth factor. It should be pointed out that there was no indication of prior activation in the control macrophage profiles, nor was there any evidence of hyporesponsiveness of macrophages in the acute (4 h) condition. It could be argued that TLR-tolerance is an essential negative regulator of deleterious inflammation, such that this program would be difficult to subvert. The dominant pattern of LPS-tolerance relative to the other TLR ligands is in line with the argument that tolerance is predominantly driven by autoregulation of NF-κB.

Our data reveals that the patterns of genes repressed during tolerance are largely associated with NF-κB dependent transcription regardless of TLR ligand, while IRF and B-ZIP motifs are over-represented in the promoters of genes that are super-induced in tolerant cells. This likely reflects the pivotal role of the NF-κB transcription factor as a driver of pro-inflammatory genes downstream of all TLRs. Previous studies have established the importance of NF-κB in promoting inflammatory gene induction (11) and tolerance (9, 21) through the differential binding of NF-κB dimers to the promoters of repressed genes. The TLR-inducible expression of NF-κB target genes relies on the transactivation domains of NF-κB dimers containing a p65(RELA) or c-REL subunit. The transcriptional repression of NF-κB target genes during tolerance requires the binding of NF-κB p50 homodimers. The NF-κB p50 subunit lacks the transactivation domain found in the p65, c-REL, and RELB subunits of NF-κB, and in the homodimeric form acts as a transcriptional repressor of NF-κB target genes. The stability of p50 homodimers is a key determinant of their repressor function and is controlled by polyubiquitination and proteasomal degradation. The IκB family member BCL-3 regulates p50 homodimer stability by inhibiting p50 ubiquitination and proteasomal degradation to form a stable DNA-bound repressor complex (9). Our data identifies elevated p50 and BCL-3 levels as a common feature of macrophages tolerized by all TLRs tested and suggests that this is a core mechanism for the repression of pro-inflammatory gene expression in TLR tolerant cells.

The repression of pro-inflammatory cytokine expression is one of the characteristic features of LPS tolerance. However, our analysis demonstrates that although each TLR ligand generally represses pro-inflammatory cytokine expression in tolerant cells, all cytokines are not universally tolerized and there is a highly diverse pattern of cytokine expression across all TLRs. A universal rule was that genes could not be tolerized if they were not first acutely induced. *Tnf* is repressed in macrophages tolerized by all of the TLR ligands tested, however, other important cytokines, such as *Il6* are repressed in cells tolerized by TLR4 and TLR3 stimulation, but not by TLR2 and TLR9 activation. Of note, *Ifnb1* is repressed in cells tolerized by ligand for TLR4 and TLR2, but not by ligands for TLR3 or TLR9. The lack of repression of *Ifnb1* expression in cells tolerized by TLR3 ligand may reflect the importance of interferons in mediating an anti-viral immune response. This data suggest that sustained expression of *Ifnb1* in the context of a viral infection may be beneficial to host immunity. Similarly, the expression of the chemokines *Cxcl9* and *Cxcl10* by TLR3 tolerized macrophages correlates with the role of these factors in CD8+ T cell recruitment to sites of viral infection, cells that have a critical anti-viral activity (23). Similarly, the re-stimulation of TLR9 or TLR2 tolerant cells induced *Il10* expression at levels comparable to the stimulation of naïve macrophages, while *Il10* expression was repressed in cells pre-treated with ligands for TLR4 and TLR3. How these TLR-specific patterns of cytokine and chemokine expression are regulated is not known, however, the differential induction of negative regulators of TLR responses by individual TLRs may provide a potential mechanism. Thus, our data indicate specific programs of TLR tolerance that are tailored toward the nature of the initiating stimulus. The immunological consequences of these specific patterns of cytokine repression will require further experimental investigation.

The differential profiles of cytokine expression in macrophages tolerized by different TLR ligands found in our analysis are also relevant to the more recently defined phenomenon of innate immune training. Innate immune training has been defined as enhanced innate host defense upon re-infection by the same or a different pathogen. Innate training is viewed as separate state to tolerance which is associated with the repression of inflammatory responses. However, our data suggest that this distinction may be too simplistic, a categorization of macrophage states following TLR activation. Our data show that while *Tnf* expression is repressed in macrophages tolerized by all TLRs tested, *Il12a* shows an expression profile characteristic of training in cells tolerized by TLR9. Indeed our data are consistent with previous studies demonstrating a protective effect of CpG treatment against infection by *L. monocytogenes* that is accompanied by sustained IL-12 production (24). It is worth noting that innate training has been largely experientially defined by the enhanced expression of a limited number of cytokines. Our data are also highly relevant to the phenomenon of cross tolerance, where ligand for one TLR can repress gene expression to subsequent re-stimulation with ligands for another TLR. To date most of the studies performed in this area have focused on a small number of cytokines, such as TNFα and IL-6. In light of our data presented here, a comprehensive transcriptomic analysis of TLR-cross tolerance is warranted in order to determine the potential relationship of cross tolerance to innate immune training. Our data caution against ascribing broad features of tolerance or training when studying TLR responses using a small number of experimental parameters. Rather, tolerance and training should be considered in a gene-specific context. This approach would likely better reflect the complex outcome of tolerization of macrophages by individual TLRs as revealed by our analysis.

In summary, this study defines the transcriptional responses of macrophages tolerized with ligands for TLR2, TLR3, TLR4, and TLR9. Our data support the concept that TLR tolerance promotes a shift away from a pro-inflammatory transcriptional response toward a response that is pro-resolution and anti-inflammatory in nature. The repression of transcription is generally associated with NF-κB target genes, while genes with IRF motifs are more likely to be super-induced in tolerant cells. However, this study also reveals the differential repression of cytokines and chemokines in macrophages tolerized by specific TLR ligands. These patterns of expression may have functional relevance to stimulus specific inflammatory responses and may also be relevant to the study of innate immune training.

## Accessions

The data is available from (GSE81291) and Stemformatics (http://www.stemformatics.org/datasets/search?ds_id=6943).

## Ethics Statement

This study was carried out in accordance with the recommendations of the Code of Practice for the Humane Killing of Animals under Schedule 1 to the UK Animals (Scientific Procedures) Act 1986, where excess tissue was used for isolation of bone marrow cells.

## Author Contributions

SB—bioinformatics, manuscript writing. CO—data generation. CW—data analysis, manuscript writing. RC—data analysis, manuscript writing.

## Conflict of Interest Statement

The authors declare that the research was conducted in the absence of any commercial or financial relationships that could be construed as a potential conflict of interest.
