# Supplementary material for: Toll-Like Receptors Drive Specific Patterns of Tolerance and Training on Restimulation of Macrophages
Source: Front Immunol. 2018 May 14;9:933. doi: 10.3389/fimmu.2018.00933 (PMC5960718; doi:10.3389/fimmu.2018.00933)
Supplement: Supplementary file 2 [file data_sheet_1.PDF]

Butcher *et al*. Toll-Like Receptors Drive Specific Patterns of Tolerance and Training on Restimulation of Macrophages

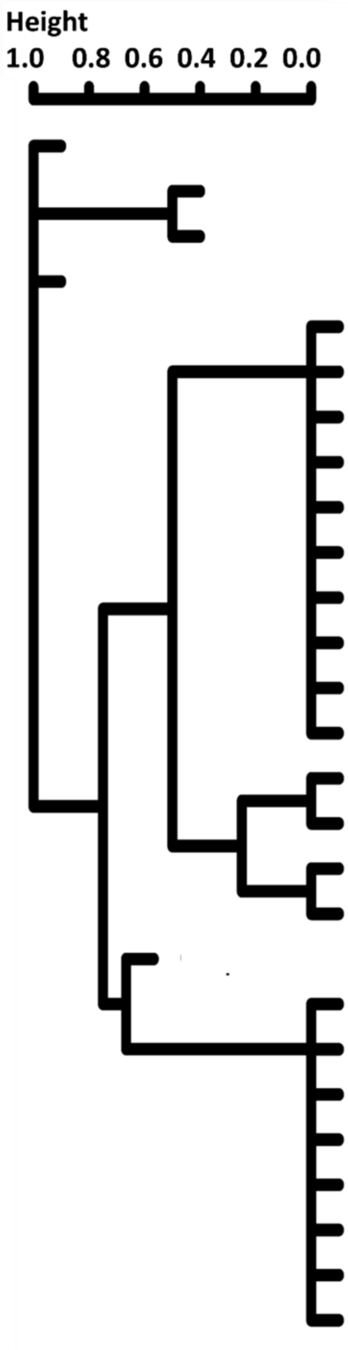

TRANSCRIPTION FACTOR (FAMILY)	MOTIF	LPS	CpG	Pam3csk4	PolyI:C
EWS:ERG-fusion<br>(ETS)	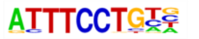				T: 16.55<br>B: 11.55%)
Stat3<br>(Stat)	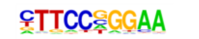			T: 15.91%<br>B: 10.10%	
STAT4<br>(Stat)	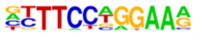			T: 17.34%<br>B: 11.73%	T: 19.35%<br>B: 13.52%
HOXB13<br>(Homeobox)	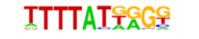		T: 15.95%<br>B: 10.36%		
PU.1:IRF8<br>(ETS:IRF)	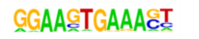	T: 10.33%<br>B: 3.04%			T: 12.12%<br>B: 3.05%
STAT1<br>(Stat)	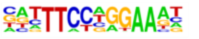	T: 8.69%<br>B: 4.62%			T: 9.79%<br>B: 4.67%
BATF<br>(bZIP)	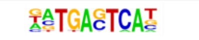	T: 11.74%<br>B: 6.02%			T: 10.49%<br>B: 6.37%
IRF:BATF<br>(IRF:bZIP)	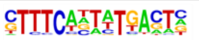	T: 3.76%<br>B: 0.93%			T: 3.26%<br>B: 1.01%
IRF4<br>(IRF)	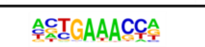	T: 11.27% B: 5.14%)			T: 12.35%<br>B:5.28%
bZIP:IRF<br>(bZIP,IRF)	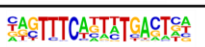	T: 10.09%<br>B: 3.99%			T: 11.19%<br>B: 4.10%
PRDM1<br>(Zf)	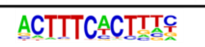	T: 15.73%<br>B: 6.72%			T: 15.85%<br>B: 6.85%
T1ISRE<br>(IRF)	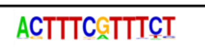	T: 2.82%<br>B: 0.16%			T: 3.50%<br>B: 0.14%
IRF2<br>(IRF)	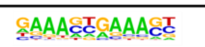	T: 12.91%<br>B: 1.86%)			T: 15.15%<br>B: 1.87%
PU.1-IRF<br>(ETS:IRF)	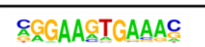	T: 29.34%<br>B: 22.08%			T: 31.24%<br>B: 22.41%
ISRE<br>(IRF)	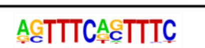	T: 11.03%<br>B: 0.99%	T: 3.64%<br>B: 1.05%		T: 13.52%<br>B: 0.95%
IRF1<br>(IRF)	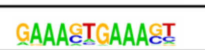	T: 12.91%<br>B: 2.05%	T: 5.69%<br>B: 2.04%		T: 15.62%<br>B: 2.04%
NFkB-p65-Rel<br>(RHD)	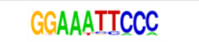	T: 3.76%<br>B:1.00%	T: 5.69%<br>B: 0.91%	T: 4.99%<br>B: 0.86%	T: 3.03%<br>B: 1.01%
NFkB-p50,p52<br>(RHD)	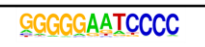	T: 5.63%<br>B: 2.35%	T: 7.06%<br>B: 2.45%	T: 5.46%<br>B: 2.48%	T: 5.13%<br>B: 2.27%
NFkB-p65<br>(RHD)	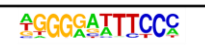	T: 11.50%<br>B: 7.43%	T: 14.81%<br>B: 7.30%	T: 12.35%<br>B: 6.94%	
Klf4<br>(Zf)	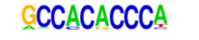	T: 23.24%<br>B: 17.80%			
Chop<br>(bZIP)	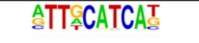	T: 4.23%<br>B: 1.90%)			
Klf9<br>(Zf)	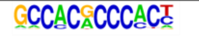	T: 30.28%<br>B: 23.62%			
Atf4<br>(bZIP)	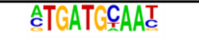	T: 5.63%<br>B: 2.59%			
Fra1<br>(bZIP)	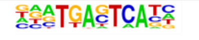	T: 9.15%<br>B: 5.06%			
Fosl2<br>(bZIP)	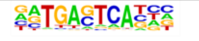	T: 6.81%<br>B: 3.26%			
Atf3<br>(bZIP)	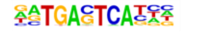	T: 10.80%<br>B: 6.04%			
AP-1<br>(bZIP)	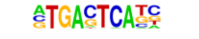	T: 11.97%<br>B: 6.97%			

Supplementary Figure 1: Transcription factor motif analysis of top 500 genes acutely regulated by TLR ligands in BMDM. Similarity tree grouping p-values across samples together. T: percent enrichment in test (top 500 acute infection) set. B: percent enrichment in background set (from all detected genes on microarray). Full transcription factor enrichment results are available at www.stemformatics.org [1].

S2A

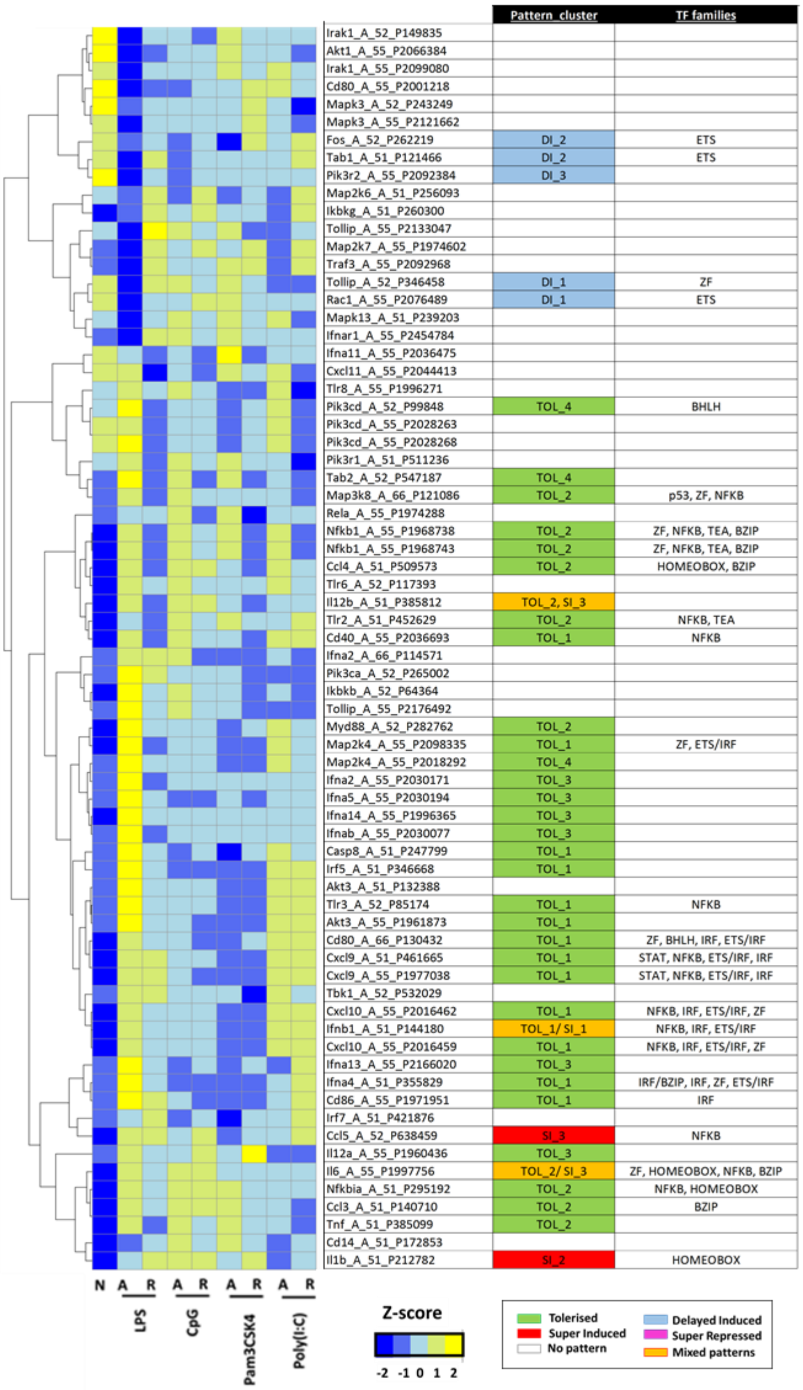

Supplementary figure 2. Hierarchical clustering of genes in the TLR pathway. (A) (Above) Heat map of probes measuring expression of TLR pathway (KEGG) [2] genes. Colour indicates row Z-score of mean log_2 expression. X axis: treatment (N: Naïve, A: acute, R: re-infection). Y-axis: Pearson correlation of z scores. Table columns (L-R): probe depicted in adjacent heatmap row, pattern/ cluster identifier (refer Figures 4, 6, S5, S6), transcription factor families (enriched in pattern cluster) found in the gene promoter. (B) (Below) KEGG [2] pathway map highlighting genes showing patterns of tolerance, super-induction, delayed induction, and super-repression.

S2B

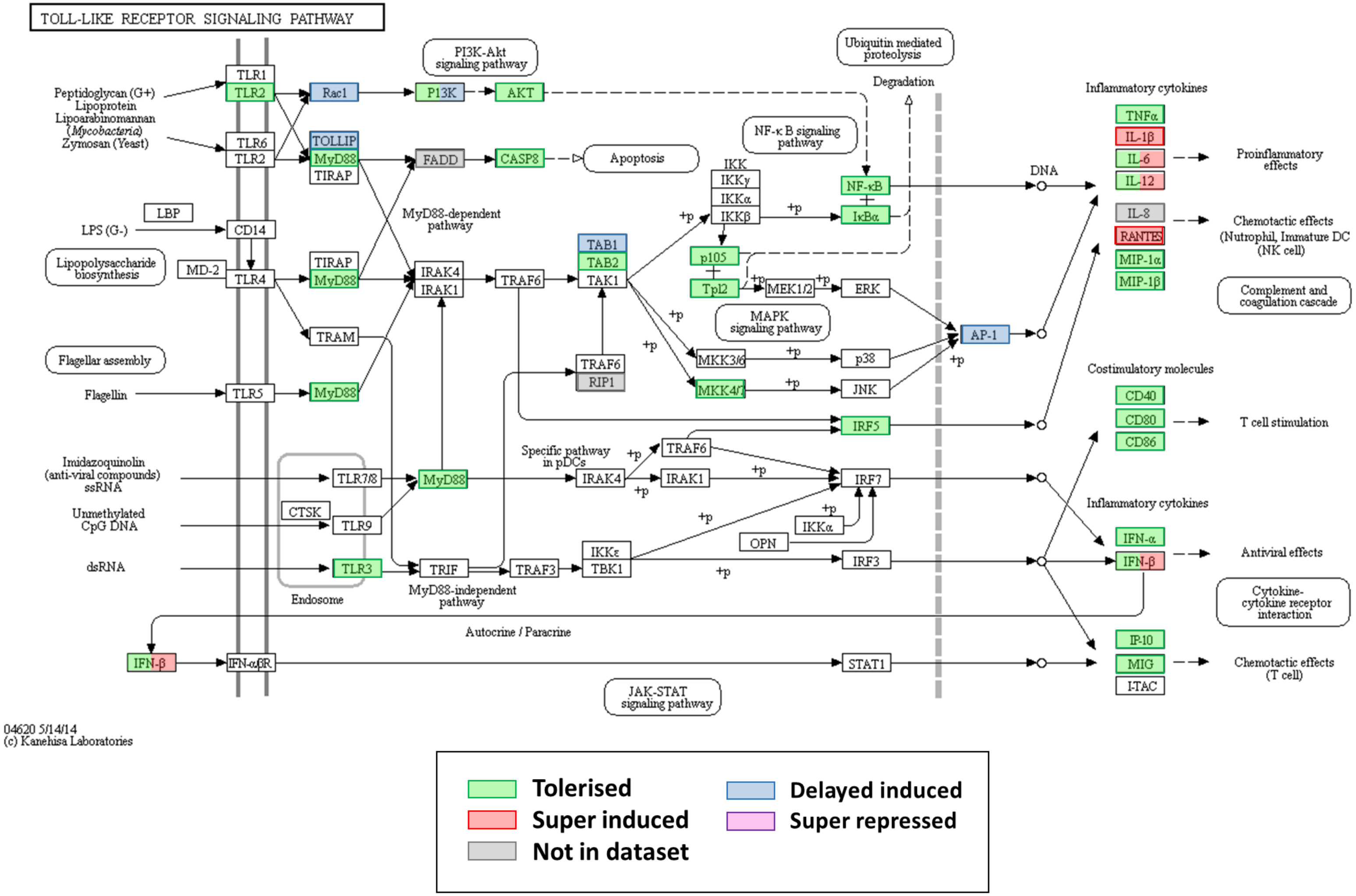
TOLL-LIKE RECEPTOR SIGNALING PATHWAY
Peptidoglycan (G+)
Lipoprotein
Lipoarabinomannan
(Mycobacteria)
Zymosan (Yeast)
TLR1
TLR2
TLR6
TLR2
Rac1
PI3K
AKT
PI3K-Akt signaling pathway
TOLLIP
MyD88
TIRAP
FADD
CASP8
Apoptosis
Ubiquitin mediated proteolysis
Degradation
NF-κB signaling pathway
IKK
IKKγ
IKKα
IKKβ
NF-κB
IκBα
DNA
LBP
LPS (G-)
CD14
Lipopolysaccharide biosynthesis
MD-2
TLR4
TIRAP
MyD88
MyD88-dependent pathway
IRAK4
IRAK1
TRAF6
TAB1
TAB2
TAK1
p105
Tpl2
MEK1/2
ERK
MAPK signaling pathway
AP-1
TRAM
TRAF6
RIP1
MKK3/6
p38
MKK4/7
JNK
Flagellar assembly
Flagellin
TLR5
MyD88
IRF5
Specific pathway in pDCs
TRAF6
Imidazoquinolin
(anti-viral compounds)
ssRNA
TLR7/8
MyD88
IRAK4
IRAK1
IRF7
Unmethylated CpG DNA
CTSK
TLR9
IKKα
OPN
dsRNA
TLR3
Endosome
TRIF
TRAF3
IKKε
TBK1
IRF3
MyD88-independent pathway
Autocrine / Paracrine
IFN-β
IFN-α/βR
STAT1
JAK-STAT signaling pathway
+p
Inflammatory cytokines
TNFα
IL-1β
IL-6
IL-12
Proinflammatory effects
IL-8
RANTES
MIP-1α
MIP-1β
Chemotactic effects
(Nutrophil, Immature DC (NK cell)
Complement and coagulation cascade
Costimulatory molecules
CD40
CD80
CD86
T cell stimulation
Inflammatory cytokines
IFN-α
IFN-β
Antiviral effects
Cytokine-cytokine receptor interaction
IP-10
MIG
I-TAC
Chemotactic effects (T cell)
04620 5/14/14
(c) Kanehisa Laboratories
Tolerised
Super induced
Not in dataset
Delayed induced
Super repressed

Butcher *et al*. Toll-Like Receptors Drive Specific Patterns of Tolerance and Training on Restimulation of Macrophages

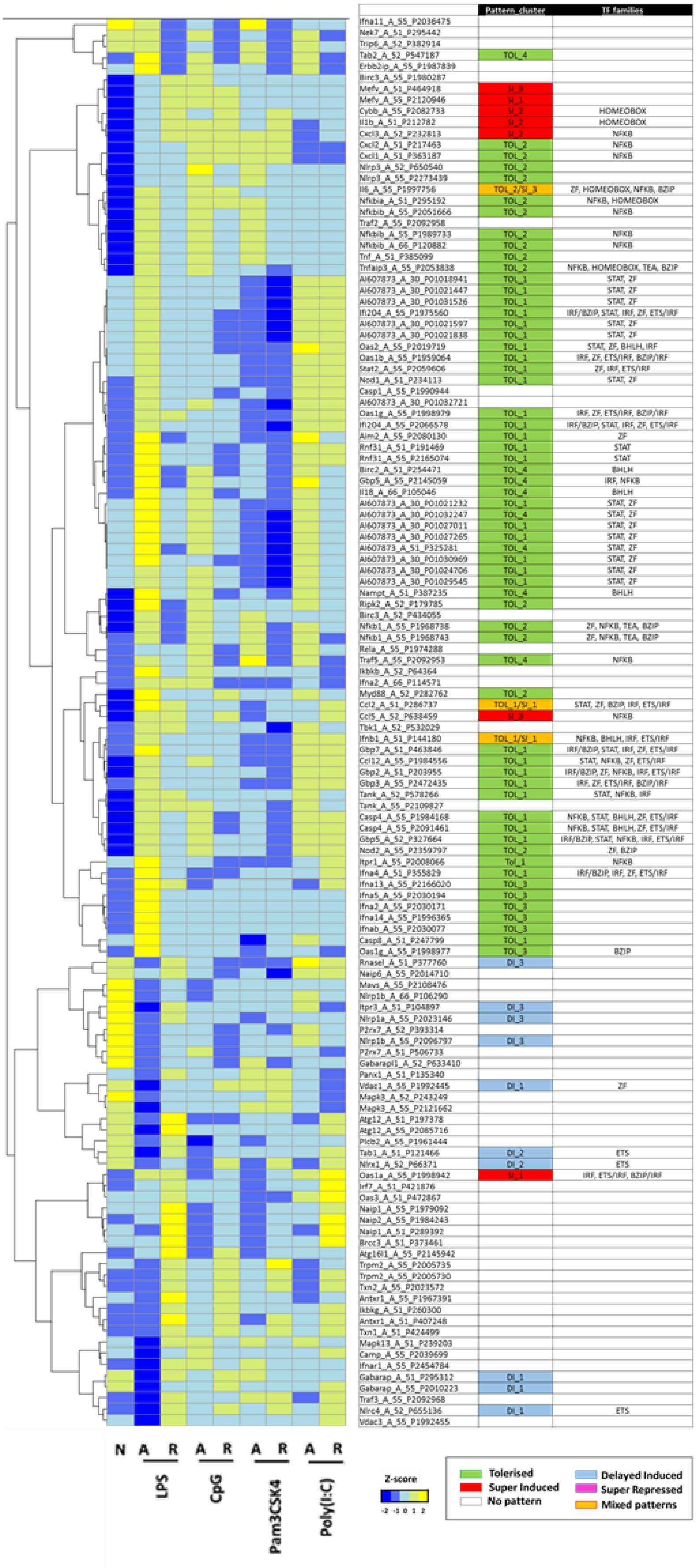

S3A

Supplementary figure 3. Hierarchical clustering of genes in the NLR pathway. (A) (Left) Heat map of probes measuring expression of NLR pathway (KEGG) [2] genes. Colour indicates row Z-score of mean $\log_2$ expression. X axis: treatment (N: Naïve, A: acute, R: re-infection). Y-axis: Pearson correlation of z scores. Table columns (L-R): probe depicted in adjacent heatmap row, pattern/ cluster identifier (refer Figures 4, 6, S5, S6), transcription factor families (enriched in pattern cluster) found in the gene promoter. (B) (Below) KEGG [2] pathway map highlighting genes showing patterns of tolerance, super-induction, delayed induction, and super-repression.

Butcher *et al*. Toll-Like Receptors Drive Specific Patterns of Tolerance and Training on Restimulation of Macrophages

S3B

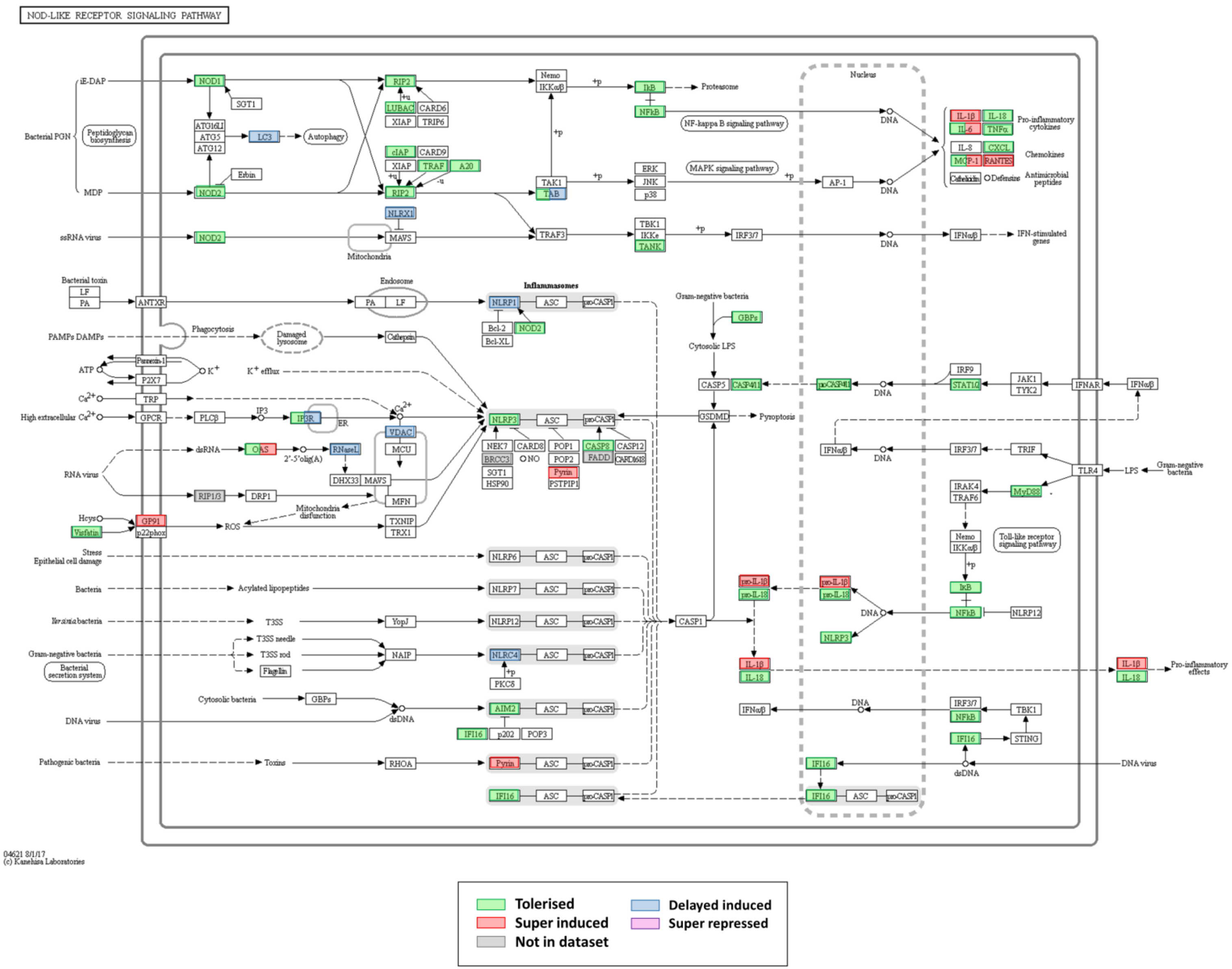

S4A

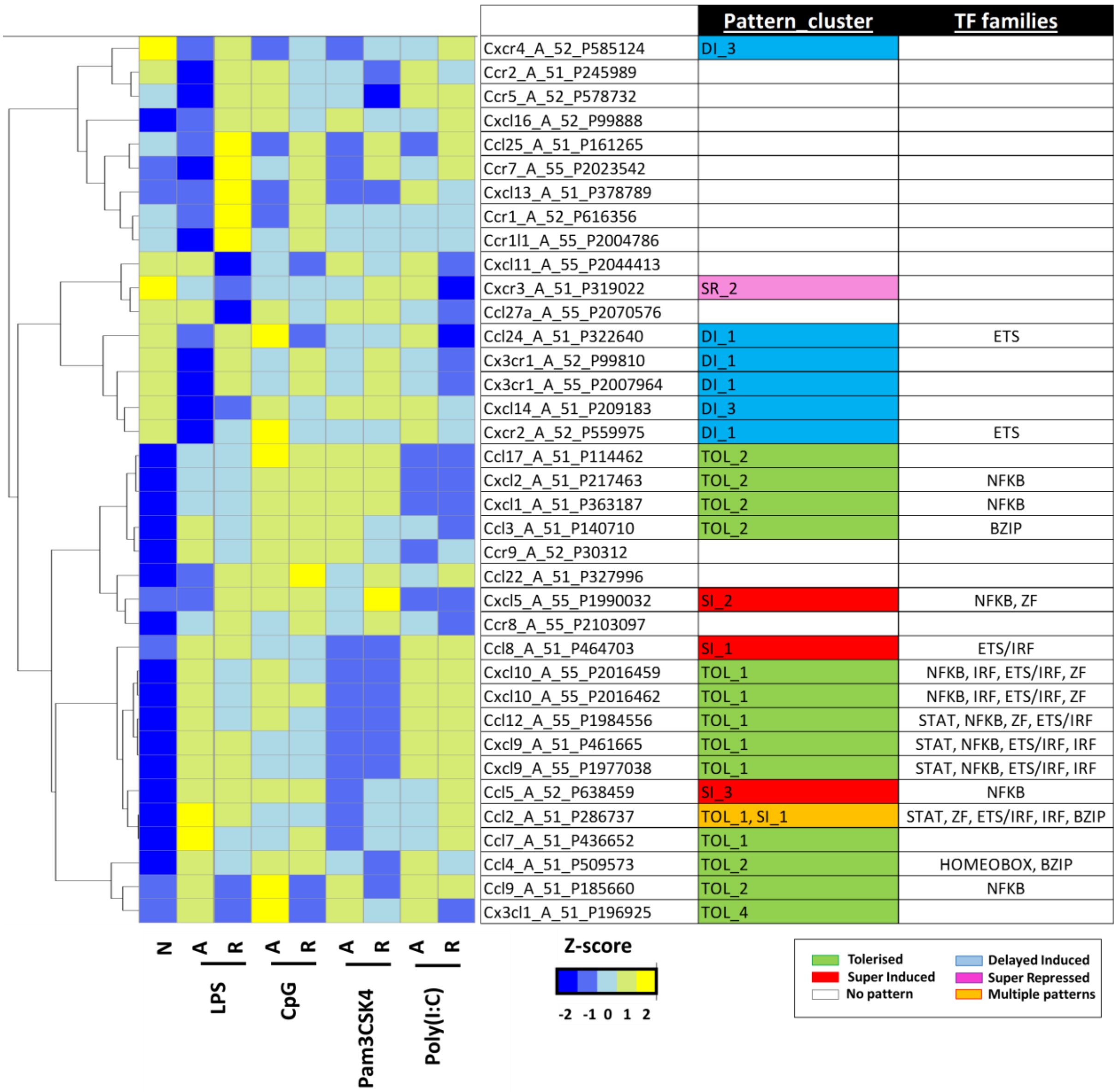

	Pattern_cluster	TF families
Cxcr4_A_52_P585124	DI_3	
Ccr2_A_51_P245989		
Ccr5_A_52_P578732		
Cxcl16_A_52_P99888		
Ccl25_A_51_P161265		
Ccr7_A_55_P2023542		
Cxcl13_A_51_P378789		
Ccr1_A_52_P616356		
Ccr1l1_A_55_P2004786		
Cxcl11_A_55_P2044413		
Cxcr3_A_51_P319022	SR_2	
Ccl27a_A_55_P2070576		
Ccl24_A_51_P322640	DI_1	ETS
Cx3cr1_A_52_P99810	DI_1	
Cx3cr1_A_55_P2007964	DI_1	
Cxcl14_A_51_P209183	DI_3	
Cxcr2_A_52_P559975	DI_1	ETS
Ccl17_A_51_P114462	TOL_2	
Cxcl2_A_51_P217463	TOL_2	NFKB
Cxcl1_A_51_P363187	TOL_2	NFKB
Ccl3_A_51_P140710	TOL_2	BZIP
Ccr9_A_52_P30312		
Ccl22_A_51_P327996		
Cxcl5_A_55_P1990032	SI_2	NFKB, ZF
Ccr8_A_55_P2103097		
Ccl8_A_51_P464703	SI_1	ETS/IRF
Cxcl10_A_55_P2016459	TOL_1	NFKB, IRF, ETS/IRF, ZF
Cxcl10_A_55_P2016462	TOL_1	NFKB, IRF, ETS/IRF, ZF
Ccl12_A_55_P1984556	TOL_1	STAT, NFKB, ZF, ETS/IRF
Cxcl9_A_51_P461665	TOL_1	STAT, NFKB, ETS/IRF, IRF
Cxcl9_A_55_P1977038	TOL_1	STAT, NFKB, ETS/IRF, IRF
Ccl5_A_52_P638459	SI_3	NFKB
Ccl2_A_51_P286737	TOL_1, SI_1	STAT, ZF, ETS/IRF, IRF, BZIP
Ccl7_A_51_P436652	TOL_1	
Ccl4_A_51_P509573	TOL_2	HOMEOBOX, BZIP
Ccl9_A_51_P185660	TOL_2	NFKB
Cx3cl1_A_51_P196925	TOL_4	

Supplementary figure 4. Hierarchical clustering of genes in the Cytokine-Cytokine Receptor Interaction pathway. (A) (Above) Heat map of probes measuring expression of Chemokines and Cytokines (B) (Below) within the Cytokine-Cytokine Receptor Interaction pathway (KEGG) [2]. Colour indicates row Z-score of mean $\log_2$ expression. X axis: treatment (N: Naïve, A: acute, R: re-infection). Y-axis: Pearson correlation of z scores. Table columns (L-R): probe depicted in adjacent heatmap row, pattern/ cluster identifier (refer Figures 4, 6, S5, S6), transcription factor families (enriched in pattern cluster) found in the gene promoter. (C) (Below) KEGG [2] pathway map highlighting genes showing patterns of tolerance, super-induction, delayed induction, and super-repression.

Butcher *et al*. Toll-Like Receptors Drive Specific Patterns of Tolerance and Training on Restimulation of Macrophages

S4B

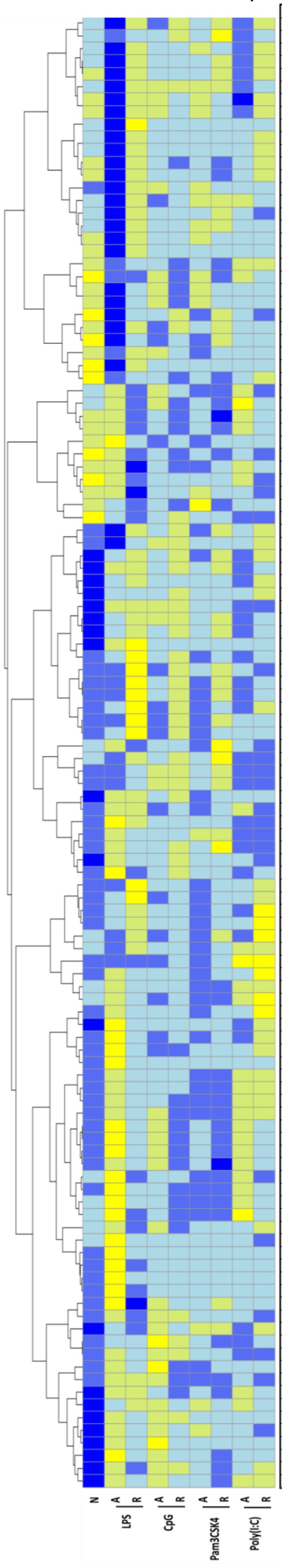

	Pattern_cluster	TF families
Ltbr_A_51_P156274		
Relt_A_51_P439299		
Ifngr2_A_52_P471395	DI_1	
Vegfa_A_52_P249424	DI_2	ZF, ETS
Vegfa_A_52_P638895	DI_1	ZF, ETS
Acvr1b_A_55_P2123957	DI_1	
Pdgfb_A_55_P1974441	DI_1	
Pdgfb_A_55_P2047310	DI_1	
Il4_A_51_P237865		
Csf2rb_A_52_P52618	DI_1	ETS
Csf2rb_A_66_P123635	DI_1	ETS
Csf2rb2_A_51_P146753		
Csf2rb2_A_55_P2034663	DI_3	
Ifnar1_A_55_P2454784		
Figf_A_51_P117739		
Pf4_A_51_P441426		
Csf1r_A_55_P2025078	DI_1	
Csf1r_A_52_P602091	DI_1	
Csf1r_A_55_P2025073	DI_1	
Il7r_A_51_P196695	DI_3	
Tgfbr1_A_55_P2137206		
Pdgfa_A_55_P2007470	DI_3	
Pdgfa_A_55_P2125588	DI_3	
Tnfrsf21_A_52_P451073		
Tnfrsf13b_A_55_P1981964	DI_2	
Il6ra_A_55_P2068892	DI_2	ETS, ZF
Tnfrsf18_A_55_P2025490	DI_3	
Acvr2b_A_55_P2012815	DI_3	
Il10rb_A_55_P2181109		
Il18rap_A_52_P517098		
Il10ra_A_51_P235984		
Pdgfc_A_52_P376360		
Bmp2_A_55_P2041961		
Bmpr1b_A_66_P136389		
Epor_A_51_P435339		
Tgfbr2_A_55_P2107288		
Tgfbr2_A_51_P450573		
Vegfb_A_52_P436628		
Ifna11_A_55_P2036475		
Lifr_A_55_P2159259	SR_1	
Il1r2_A_51_P470079		
Il28ra_A_52_P621588		
Il17ra_A_51_P348280		
Vegfc_A_51_P433870		
Inhba_A_51_P239750	SI_3	bZIP
Tnfrsf14_A_55_P2085915		
Pdgfrb_A_55_P1957249	TOL_2	HOMEOBOX, BZIP
Il1b_A_51_P212782	SI_2	HOMEOBOX
Il1a_A_52_P100926	SI_2	NFKB, HOMEOBOX
Met_A_51_P103929	SI_3	bZIP
Cd27_A_55_P2164428		
Flt3_A_66_P116412		
Il1r1_A_51_P271503		
Il4ra_A_55_P1966189		
Il23r_A_66_P116173	SI_2	
Tnfrsf9_A_55_P2029061		
Il15ra_A_55_P2060707		
Egfr_A_55_P1956862		
Ppbp_A_51_P428372		
Csf3_A_51_P317176	SI_2	NFKB
Il4ra_A_55_P2017789		
Acvr1_A_55_P2102693		
Acvr1_A_55_P2159710		
Inhbe_A_51_P357735		
Egfr_A_52_P106259	TOL_3	
Il12a_A_55_P1960436	TOL_3	
Il1rap_A_52_P467232	TOL_2	ZF
Il2ra_A_55_P1980796	TOL_3	
Il12rb1_A_55_P2034032		
Il12rb1_A_55_P2034033		
Osm_A_51_P291361		
Il2rg_A_66_P113749		
Tgfb3_A_51_P124748		
Il21r_A_55_P2071447		
Cntfr_A_55_P2133007	TOL_1	
Ghr_A_51_P499854	TOL_1	
Kdr_A_51_P316553	TOL_1	ZF, NFKB
Inhbb_A_55_P2096422	TOL_1	ZF, ETS/IRF
Tnfrsf11b_A_52_P106620	TOL_1	BHLH, ZF, ETS/IRF
Tnfrsf14_A_55_P2046661		
Ifna13_A_55_P2166020	TOL_3	
Ifna4_A_51_P355829	TOL_1	IRF/BZIP, IRF, ZF, ETS/IRF
Lep_A_52_P374882		
Ifnb1_A_51_P144180	TOL_1, SI_1	NFKB, BHLH, IRF, ETS/IRF
Tnfsf10_A_55_P2018017	TOL_1	IRF, ETS/IRF, ZF, BZIP/IRF
Il15ra_A_55_P2041738	TOL_1	IRF, ZF
Il13ra1_A_52_P552194	TOL_1	ZF, ETS/IRF
Il24_A_55_P2127215		
Il18_A_66_P105046	TOL_4	BHLH
Csf1_A_51_P195506	TOL_1	NFKB, ZF
Il15_A_52_P15461	TOL_1	ZF, NFKB, BHLH
Tnfrsf1a_A_52_P192426	TOL_4	
Tnfsf18_A_55_P2075553	TOL_4	NFKB
Kitl_A_55_P2001494	TOL_4	NFKB
Tnfsf8_A_55_P2179463		
Tslp_A_51_P463003		
Il17d_A_55_P1980051	TOL_3	
Ifna5_A_55_P2030194	TOL_3	
Ifna2_A_55_P2030171	TOL_3	
Ifna14_A_55_P1996365	TOL_3	
Ifnab_A_55_P2030077	TOL_3	
Ltb_A_51_P302358	TOL_2	ZF, NFKB
Il10_A_51_P430766	TOL_2	NFKB
Flt4_A_51_P179531		
Il19_A_55_P2001489	TOL_2	
Il19_A_55_P2128144	TOL_2	
Acvr2a_A_52_P664656		
Ifna2_A_66_P114571		
Il20rb_A_55_P2023294	TOL_1	ZF
Fas_A_55_P2091676		
Il6_A_55_P1997756	TOL_2, SI_3	ZF, HOMEOBOX, NFKB, BZIP
Tnf_A_51_P385099	TOL_2	
Tnfsf9_A_55_P2010038	TOL_2	HOMEOBOX
Tnfsf15_A_55_P2182452	TOL_4	
Il12b_A_51_P385812	TOL_2, SI_3	
Cd40_A_55_P2036693	TOL_1	NFKB

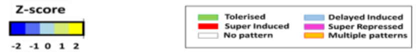

S4C

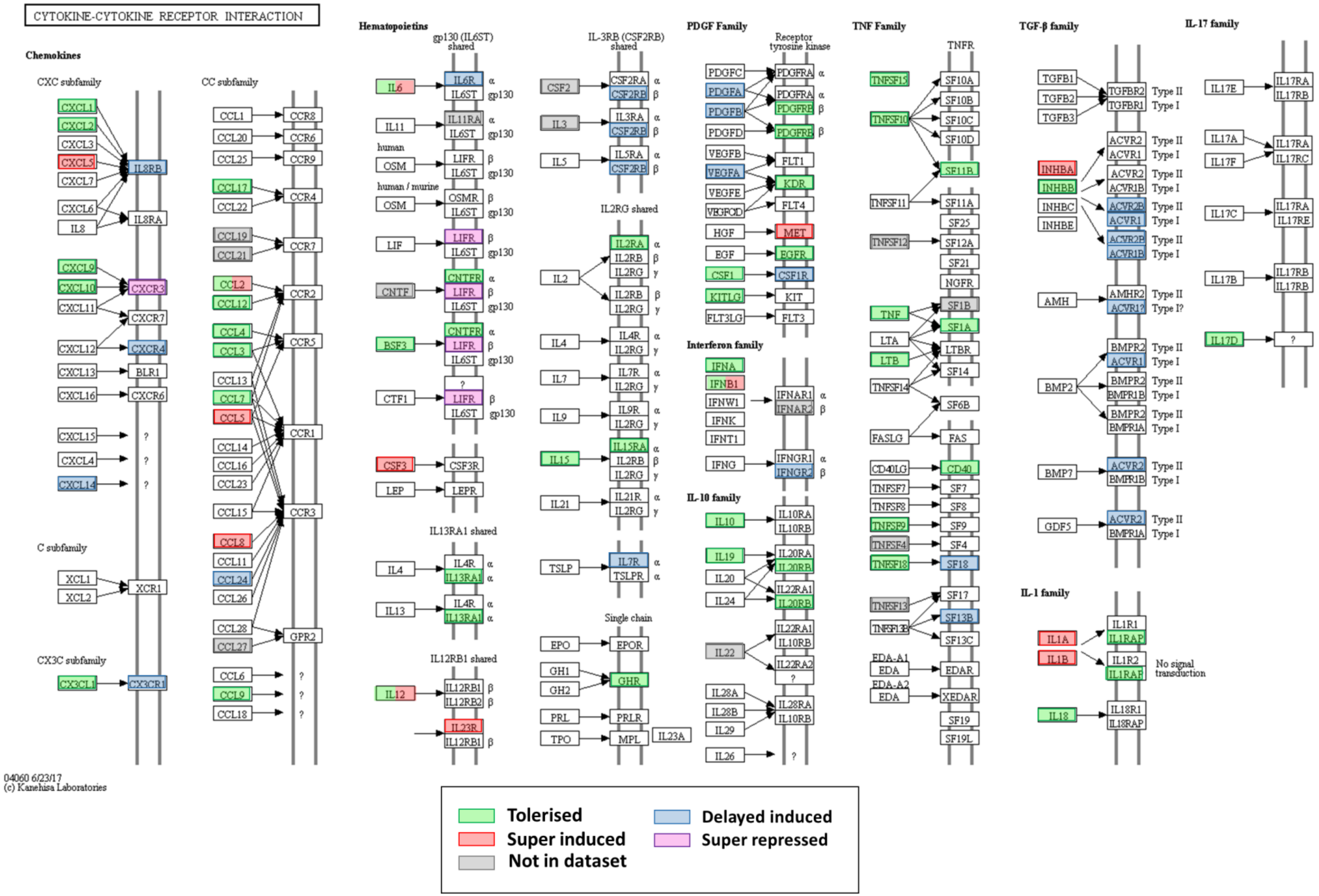

CYTOKINE-CYTOKINE RECEPTOR INTERACTION
Chemokines
CXC subfamily
CXCL1
CXCL2
CXCL3
CXCL5
CXCL7
CXCL6
IL8
IL8RB
IL8RA
CXCL9
CXCL10
CXCL11
CXCR3
CXCR7
CXCL12
CXCR4
CXCL13
BLR1
CXCL16
CXCR6
CXCL15
CXCL4
CXCL14
C subfamily
XCL1
XCL2
XCR1
CX3C subfamily
CX3CL1
CX3CR1
CC subfamily
CCL1
CCR8
CCL20
CCR6
CCL25
CCR9
CCL17
CCL22
CCR4
CCL19
CCL21
CCR7
CCL2
CCL12
CCR2
CCL4
CCL3
CCR5
CCL13
CCL7
CCL5
CCR1
CCL14
CCL16
CCL23
CCL15
CCR3
CCL8
CCL11
CCL24
CCL26
CCL28
CCL27
GPR2
CCL6
CCL9
CCL18
Hematopoietins
gp130 (IL6ST) shared
IL6
IL6R
IL6ST
IL11
IL11RA
human
OSM
LIFR
human / murine
OSMR
LIF
CNTF
CNTFR
BSF3
CTF1
IL13RA1 shared
IL4
IL4R
IL13RA1
IL13
IL12RB1 shared
IL12
IL12RB1
IL12RB2
IL23R
CSF3
CSF3R
LEP
LEPR
IL-3RB (CSF2RB) shared
CSF2
CSF2RA
CSF2RB
IL3
IL3RA
IL5
IL5RA
IL2RG shared
IL2
IL2RA
IL2RB
IL2RG
IL4R
IL7
IL7R
IL9
IL9R
IL15
IL15RA
IL21
IL21R
TSLP
TSLPR
Single chain
EPO
EPOR
GH1
GH2
GHR
PRL
PRLR
TPO
MPL
IL23A
PDGF Family
Receptor tyrosine kinase
PDGFC
PDGFA
PDGFB
PDGFD
PDGFRA
PDGFRB
VEGFB
VEGFA
VEGFE
VEGFC/D
FLT1
KDR
FLT4
HGF
MET
EGF
EGFR
CSF1
CSF1R
KITLG
KIT
FLT3LG
FLT3
Interferon family
IFNA
IFNB1
IFNW1
IFNK
IFNT1
IFNAR1
IFNAR2
IFNG
IFNGR1
IFNGR2
IL-10 family
IL10
IL10RA
IL10RB
IL19
IL20
IL24
IL20RA
IL20RB
IL22RA1
IL22
IL22RA2
IL28A
IL28B
IL29
IL28RA
IL26
TNF Family
TNFR
TNFSF15
TNFSF10
TNFSF11
TNFSF12
TNF
LTA
LTB
TNFSF14
FASLG
CD40LG
TNFSF7
TNFSF8
TNFSF9
TNFSF4
TNFSF18
TNFSF13
TNFSF13B
EDA-A1
EDA-A2
EDA
SF10A
SF10B
SF10C
SF10D
SF11B
SF11A
SF25
SF12A
SF21
NGFR
SF1B
SF1A
LTBR
SF14
SF6B
FAS
CD40
SF7
SF8
SF9
SF4
SF18
SF17
SF13B
SF13C
EDAR
XEDAR
SF19
SF19L
TGF-β family
TGFB1
TGFB2
TGFB3
TGFBR2
TGFBR1
INHBA
INHBB
INHBC
INHBE
ACVR2
ACVR1
ACVR1B
ACVR2B
AMH
AMHR2
BMP2
BMPR2
BMPR1B
BMPR1A
BMP7
GDF5
Type II
Type I
IL-1 family
IL1A
IL1B
IL1R1
IL1RAP
IL1R2
No signal transduction
IL18
IL18R1
IL18RAP
IL-17 family
IL17E
IL17RA
IL17RB
IL17A
IL17F
IL17RC
IL17C
IL17RE
IL17B
IL17D
04060 6/23/17
(c) Kanehisa Laboratories
Tolerised
Super induced
Not in dataset
Delayed induced
Super repressed

S5

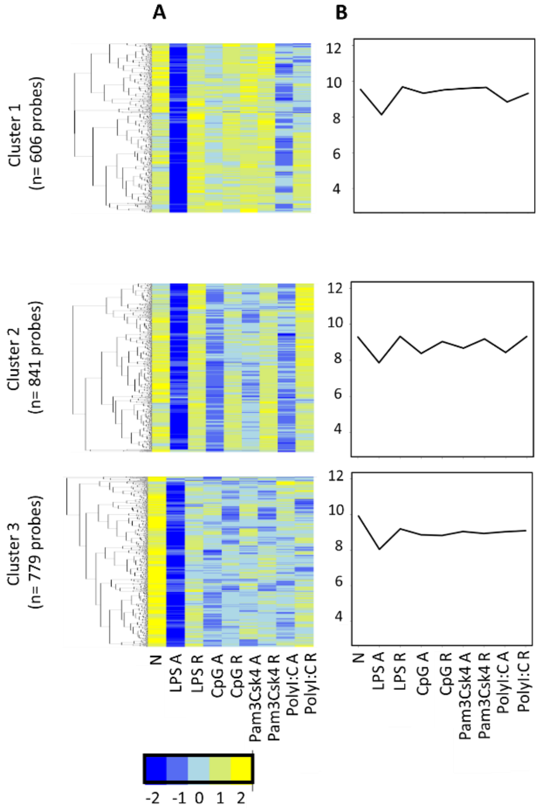

C

Top pathway themes	Maximum corrected p-value
Cellular response to stress	p < 0.034
Carbohydrate metabolism	p < 0.025
NGF signalling	p < 0.038
Vesicle trafficking	p < 0.027
Lipid metabolism	p < 0.035
Mitochondrial translation, biogenesis and maintenance	p < 0.003
DNA repair	p < 0.029
NO metabolism	p < 0.027
RNA Pol II Transcription	p < 0.049
Vitamin metabolism	p < 0.036
FCGR dependent phagocytosis	p < 0.233
Lipid Metabolism	p < 0.365

D

TF/ TF family	Motif	Motif q (p)	Example genes with motif
Sp1(Zf)	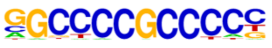	0.025 (1E-04)	Arrb2, Cebpa, Kdm4b, Klf16, Map3k2, Map4k5, Mapkapk3, Thap4, Tollip
EHF(ETS)	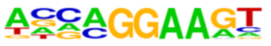	0.075 (1E-03)	Ccdc115, Ccl24, Csf2rb, Cxcr2, Irf2bp1, Nfatc1, Nlrc4, Tnfaip1
ETS Family (ETS)	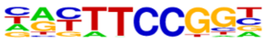	0.000 (1E-17)	B3galt4, Hdac7, Ill6ra, Irf2bpl, Sirt3, Tgfbi, Trim65
Sp1(Zf)	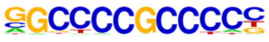	0.011 (1E-03)	Akap10, Arrdc2, Bmf, Cactin, Casp9, Erlin1, Usp20
ZFX/ZNF711 (ZF)	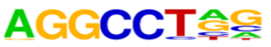	0.027 (1E-02)	Casp9, Hist2h2be, Htr2b, Prkar1b, Ncor2, Usp2
GFY-Staf	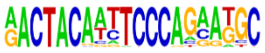	0.011 (1E-04)	Ccdc191, Zfp111, Zfp354c, Zfp358, Zfp420, Zfp128, Zfp551, Zfp444, Zfp637, Zfp661
GFY	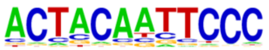	0.097 (1E-03)	Ccdc191, L3mbtl2, Mcpt8, Snapin, Zfp111, Zfp354c, Zfp358, Zfp420, Zfp128, Zfp551, Zfp444, Zfp637, Zfp661

Figure S5. Probes that demonstrate transient repression.

Probes showing transient repression in at least one infection were clustered using PAM. **(A)** Heat maps for each cluster. Colour indicates row z-score of mean log_2 expression. X axis: treatment (N: Naïve, A: acute, R: re-infection). Y-axis: Pearson correlation of z scores. **(B)** Mean log_2 expression pattern for all probes in each cluster (y-axis) per condition (x-axis). **(C)** Significantly enriched pathways, grouped thematically. Maximum adjusted p-value for all pathways significantly enriched in that theme are shown. **(D)** Transcription factor binding motifs enriched in each cluster. Motif logos and adjusted p-values are representative for each transcription factor family. Full transcription factor enrichment results are available at www.stemformatics.org [1]

S6

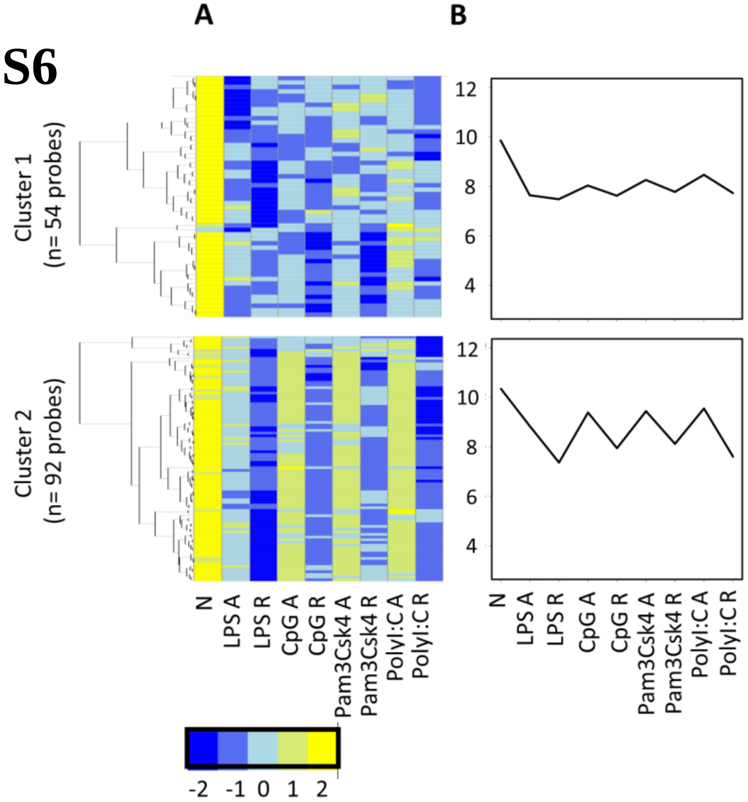

C

Top pathway themes	Maximum corrected p-value
Metabolism (vitamins)	0.029
DNA Repair	0.015

Cell division	0.033

S6: Probes that demonstrate consistent repression.

Probes showing super-repression in at least one infection were clustered using PAM. **(A)** Heat maps for each cluster. Colour indicates row z-score of mean $\log_2$ expression. X axis: treatment (N: Naïve, A: acute, R: re-infection). Y-axis: Pearson correlation of z scores. **(B)** Mean $\log_2$ expression pattern for all probes in each cluster (y-axis) per condition (x-axis). **(C)** Significantly enriched pathways, grouped thematically. Maximum adjusted p-value for all pathways significantly enriched in that theme are shown.

LPS	CpG	Pam3CSK4	Poly (I:C)
**Innate Immune Response**<br>11% (87/771); p = 7.95E-35	**Innate Immune Response**<br>9% (71/771); p = 8.36E-22	**Innate Immune Response**<br>7% (55/771); p = 7.03E-12	**Innate Immune Response**<br>11% (81/771); p=2.37E-30
**Defence Response to Virus**<br>19% (26/138); p = 2.20E-14	**Cellular Response to LPS**<br>22% (20/93), p = 1.27E-11	**Cellular Response to LPS**<br>16% (15/93); p = 9.10E-07	**Defence Response to Virus**<br>22% (32/138); p = 2.88E-21
**Cellular Response to IFN-beta**<br>59% (13/22); p = 5.99E-14	**Inflammatory Response**<br>12% (29/249); p = 1.38E-10	**Inflammatory Response**<br>9% (23/249); p = 2.45E-06	**Cellular Response to IFN-beta**<br>59% (13/22); p = 4.55E-14
**Immune Response**<br>12% (28/237); p = 1.30E-10	**Immune Response**<br>11% (27/237); p = 1.29E-09	**Response to LPS**<br>11% (18/162); p = 5.67E-06	**Response to Virus**<br>23% (21/90); p = 1.08E-13
**Response to Virus**<br>20% (18/90); p = 2.55E-10	**Response to LPS**<br>14% (22/162); p = 3.66E-09	**Positive Regulation of NF-kappaB activity**<br>14% (14/98); p = 8.30E-06	**Negative Regulation of Viral Replication**<br>46% (12/26); p = 2.37E-11

Table S2: Top 5 GO terms (biological process) enriched for each set of TLR-ligand activated genes. Enrichment for each term is shown by the proportion of the GO list overlapping the TLR-induced gene lists, the number of terms in the TLR gene list /the total number of terms in the GO category. Significance of enrichment indicated by an adjusted p-value. Full list available in Table S1.

REFERENCES

1. Wells CA, Mosbergen R, Korn O, Choi J, Seidenman N, Matigian NA, et al. Stemformatics: visualisation and sharing of stem cell gene expression. Stem Cell Res (2013) 10(3):387–95. doi:10.1016/j.scr.2012.12.003
2. Kanehisa, Furumichi, M., Tanabe, M., Sato, Y., and Morishima, K.; KEGG: new perspectives on genomes, pathways, diseases and drugs. Nucleic Acids Res (2017) 45, D353-D361. doi: 10.1093/nar/gkw1092
